# Electronic Structure and Transformation of Dinitrosyl Iron Complexes (DNICs) Regulated by Redox Non-Innocent Imino-Substituted Phenoxide Ligand

**DOI:** 10.1021/acs.inorgchem.3c03367

**Published:** 2024-01-23

**Authors:** Wun-Yan Wu, Wei-Yuan Zheng, Wei-Ting Chen, Fu-Te Tsai, Ming-Li Tsai, Chih-Wen Pao, Jeng-Lung Chen, Wen-Feng Liaw

**Affiliations:** † Department of Chemistry, National Tsing Hua University, Hsinchu 30013, Taiwan; ‡ Department of Chemistry, National Sun Yat-sen University, Kaohsiung 80424, Taiwan; § National Synchrotron Radiation Research Center, Hsinchu 30013, Taiwan

## Abstract

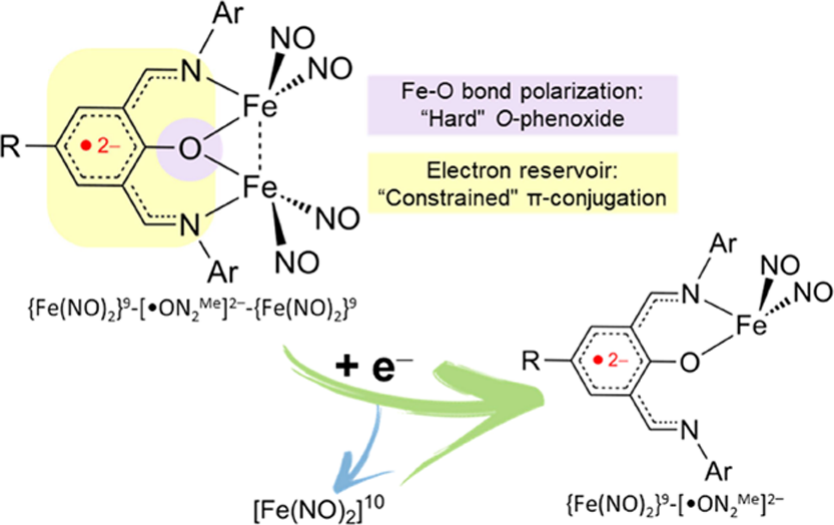

The coupled NO-vibrational peaks [IR ν_NO_ 1775 s, 1716 vs, 1668 vs cm^–1^ (THF)] between two adjacent [Fe(NO)_2_] groups implicate the electron delocalization nature of the singly *O*-phenoxide-bridged dinuclear dinitrosyliron complex (DNIC) [Fe(NO)_2_(μ-ON_2_^Me^)Fe(NO)_2_] (**1**). Electronic interplay between [Fe(NO)_2_] units and [ON_2_^Me^]^−^ ligand in DNIC **1** rationalizes that “hard” *O*-phenoxide moiety polarizes iron center(s) of [Fe(NO)_2_] unit(s) to enforce a “constrained” π-conjugation system acting as an electron reservoir to bestow the spin-frustrated {Fe(NO)_2_}^9^-{Fe(NO)_2_}^9^-[^·^ON_2_^Me^]^2–^ electron configuration (*S*_total_ = 1/2). This system plays a crucial role in facilitating the ligand-based redox interconversion, working in harmony to control the storage and redox-triggered transport of the [Fe(NO)_2_]^10^ unit, while preserving the {Fe(NO)_2_}^9^ core in DNICs {Fe(NO)_2_}^9^-[^·^ON_2_^Me^]^2–^ [K-18-crown-6-ether)][(ON_2_^Me^)Fe(NO)_2_] (**2**) and {Fe(NO)_2_}^9^-[^·^ON_2_^Me^] [(ON_2_^Me^)Fe(NO)_2_][PF_6_] (**3**). Electrochemical studies suggest that the redox interconversion among [{Fe(NO)_2_}^9^-[^·^ON_2_^Me^]^2–^] DNIC **3** ↔ [{Fe(NO)_2_}^9^-[ON_2_^Me^]^−^] ↔ [{Fe(NO)_2_}^9^-[^·^ON_2_^Me^]] DNIC **2** are kinetically feasible, corroborated by the redox shuttle between O-bridged dimerized [(μ-ON^Me^)_2_Fe_2_(NO)_4_] (**4**) and [K-18-crown-6-ether)][(ON^Me^)Fe(NO)_2_] (**5**). In parallel with this finding, the electronic structures of [{Fe(NO)_2_}^9^-{Fe(NO)_2_}^9^-[^·^ON_2_^Me^]^2–^] DNIC **1**, [{Fe(NO)_2_}^9^-[^·^ON_2_^Me^]^2–^] DNIC **2**, [{Fe(NO)_2_}^9^-[^·^ON_2_^Me^]] DNIC **3**, [{Fe(NO)_2_}^9^-[ON^Me^]^−^]_2_ DNIC **4,** and [{Fe(NO)_2_}^9^-[^·^ON^Me^]^2–^] DNIC **5** are evidenced by EPR, SQUID, and Fe K-edge pre-edge analyses, respectively.

## Abstract 2

The redox-induced extrusion of the [Fe(NO)_2_]^10^ unit along with the formation of [{Fe(NO)_2_}^9^-[^·^ON_2_^Me^]^2−^] DNIC [K-18-crown-6-ether)][(ON_2_^Me^)Fe(NO)_2_] was triggered by one-electron reduction of fully electronically delocalized [{Fe(NO)_2_}^9^-{Fe(NO)_2_}^9^-[^·^ON_2_^Me^]^2−^] DNIC [Fe(NO)_2_(μ-ON_2_^Me^)Fe(NO)_2_].

## 1 Introduction

Nitric oxide and tyrosine radical are two central endogenous radicals that are known to modulate versatile biological functions and enzymatic reactions. To harness the highly reactive nitric oxide radical without immediately generating detrimental reactive nitrogen species (RNS),^1^ nature has evolved *S*-nitrosothiol (RSNO) and dinitrosyl iron complexes (DNICs) acting as NO storage and transport vehicles to achieve homeostatic regulations of the NO concentration required for precisely exerting physiological functions including vasodilation, smooth muscle relaxation, inhibition of platelet aggregation, memory formation/learning processes in neurons, and cellular proliferation/differentiation/apoptosis.^2−10^ In contrast to the dynamic nature of NO homeostasis under pathogenic and physiological conditions, the tyrosyl radical (Ẏ) within proteins has been found to play vital roles in several enzymatic reactions associated with the conversion of ribonucleotides to 2′-deoxyribonucleotides via ribonucleotide reductase (RNR) for DNA replication/repair and cell survival,^11^ electron delivery to the O_2_-evolving complex in Photosystem II,^12,13^ electron/proton shuttling for the reduction of O_2_ to water of cytochrome *c* oxidase in the respiratory chain, and two-electron oxidation of alcohols to aldehydes of a fungal enzyme galactose oxidase (GAO).^14−16^

The advanced spectroscopic methods including ENDOR, EPR, IR, UV–vis, Mössbauer, and NRVS unveil the concomitant formation of {Fe(NO)_2_}^9^ DNIC and Roussin’s red ester (RRE) via nitrosylation of nonheme Fe proteins/model compounds.^3,6,17−27^ In a model study, biomimetic {Fe(NO)_2_}^9^ DNIC derived from nitrosylation of [Fe–S] clusters or nitrosylation of iron in the presence of cysteine/reduced glutathione was validated as [(RS)_2_Fe(NO)_2_]^−^ by the distinctive EPR signal at *g* = 2.03.^28^ Over the past decades, the in-depth biomimetic studies provide unprecedented molecular insights resulting from the developments of synthetic strategies and structural characterizations on a series of mononuclear classic four-coordinate DNICs, nonclassic five-coordinate DNICs, and binuclear DNICs coordinated/bridged with biologically relevant ligands.^29,30^ Specifically, the study concludes that both mononuclear and binuclear DNICs containing S/N/O donor sets dictate the thermodynamic stability/kinetic robustness of [Fe(NO)_2_]^9/10^ electronic configurations as well as modulate the oxidation states of coordinated NO moiety for the designated chemical reactivity.^29,31−44^ In translating molecular insights obtained from this fundamental research into real-world applications, significant progress has been made on [Fe(NO)_2_] motif serving as a functional building block for promoting electrochemical H_2_ evolution from water,^45^ CO_2_ valorization via C–C bond coupling under ambient condition,^46^ and controlled/targeted delivery of NO for biomedical treatments associated with versatile diseases.^2−8,10,17−19,26,30,47−54^

**Scheme 1:**
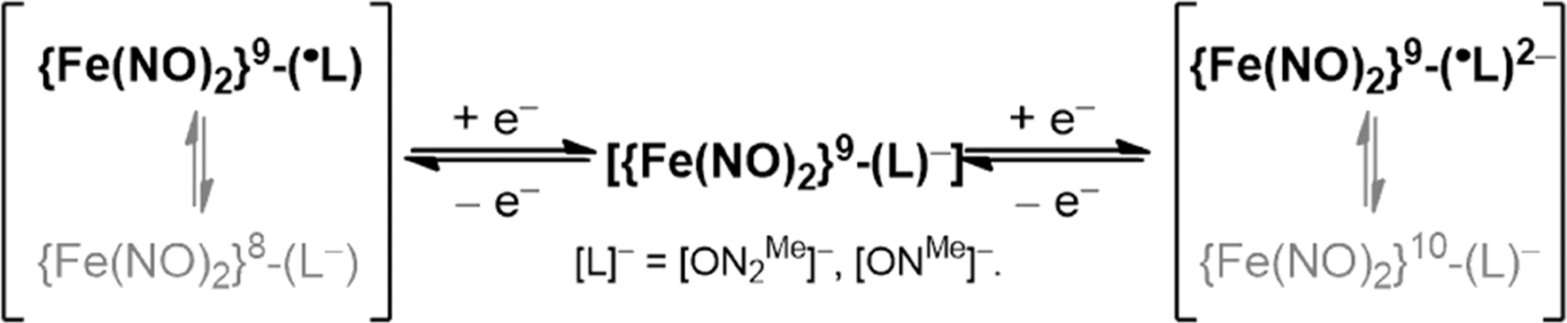
Redox Capacity of the [ON_2_^Me^]^**–**^-Coordinate [Fe(NO)_2_] Motif

Recently, {Fe(NO)_2_}^9^ DNIC [(NO)_2_Fe(N(Mes)(TMS))_2_]^−^ (Mes = mesityl, TMS = trimethylsilane) with the noninnocent [(N(Mes)(TMS))]^−^ coordinated ligands has been used to extend the redox capacity to the [{Fe(NO)_2_}^9^-(N(Mes)(TMS))^−^(N(Mes)(TMS))^·^] electronic level with a retained {Fe(NO)_2_}^9^ electronic configuration upon one-electron oxidation of DNIC [(NO)_2_Fe(N(Mes)(TMS))_2_]^−^.^32,55,56^ In addition, redox-active α-/β-imino substituent ligands have demonstrated their feature as electron reservoirs capable of undergoing multielectron transformations in metal complexes.^57−72^ The reduction of the redox-active α-diimine-incorporated {Fe(NO)_2_}^10^ DNIC [(NO)_2_Fe(^R^DDB)] (^R^DDB = N,*N*′-bis(2,6-dialkylphenyl)-1,4-diaza-2,3-dimethyl- 1,3-butadiene) with the highly covalent {Fe(NO)_2_}^10^ core led to the formation of [{Fe(NO)_2_}^10^-[^R^DDB]^·–^] DNIC [(NO)_2_Fe(^R^DDB)]^−^ via the ^R^DDB ligand accommodating excess electron density.^73^ The extensive studies of the redox interconversion among [{Fe(NO)_2_}^9^-[^R^DDB]]^+^ ↔ [{Fe(NO)_2_}^10^-[^R^DDB]] ↔ [{Fe(NO)_2_}^10^-[^R^DDB]^·–^] electronic states inspired the utilization of the redox-active α-diimine and chelating amine to assemble DNIC [(^R^DDB)Fe(NO)_2_] and [(PMDTA)Fe(NO)_2_] acting as molecular electrocatalysts for hydrogen evolution from water.^45,73^ Despite the electronic structures and bonding interactions of {Fe(NO)_2_}^9/10^ chelated with redox-active imine-/amide-based ligands have been well elucidated, the interplay between [Fe(NO)_2_] motif and the biologically relevant phenoxide ligands has not been explored yet. In this study, we reported the synthesis and characterization of the electronically delocalized, singly *O*-phenoxide-bridged dinuclear DNIC [Fe(NO)_2_(μ-ON_2_^Me^)Fe(NO)_2_] (**1**) (HON_2_^Me^ = 4-*tert*-butyl-2,6-bis(2,6-dimethylphenyl-iminomethyl)phenol). With the aid of hard/π-conjugated redox-active ligand [ON_2_^Me^]^−^, [Fe(NO)_2_] units are polarized and thus complex **1** is endowed with the [{Fe(NO)_2_}^9^-{Fe(NO)_2_}^9^-[^·^ON_2_^Me^]^2–^] electronic structure. In particular, the redox capacity of the [ON_2_^Me^]^**–**^-coordinate [Fe(NO)_2_] motif extends to the multiple electronic levels [{Fe(NO)_2_}^9^-[^·^L]^2–^] [(ON_2_^Me^)Fe(NO)_2_]^**–**^ (**2**) ↔ [{Fe(NO)_2_}^9^-[L]^−^] [(ON_2_^Me^)Fe(NO)_2_] ↔ [{Fe(NO)_2_}^9^-[^·^L]] [(ON_2_^Me^)Fe(NO)_2_]^+^ (**3**) along with the liberation of the [Fe(NO)_2_]^10^ dinitrosyl iron unit (DNIU), triggered by chemical redox treatments (Scheme 1). This finding may signify the biological relevance to the interplay between [Fe(NO)_2_] motif and phenolate/phenoxyl under the redox processes.

## 2 Results and Discussion

### 2.1 Synthesis and Characterization of Electronically Delocalized {Fe(NO)_2_}^9^-{Fe(NO)_2_}^9^-[^·^ON_2_^Me^]^2–^ DNIC [(μ-ON_2_^Me^)Fe_2_(NO)_4_] (**1**)

**Scheme 2:**
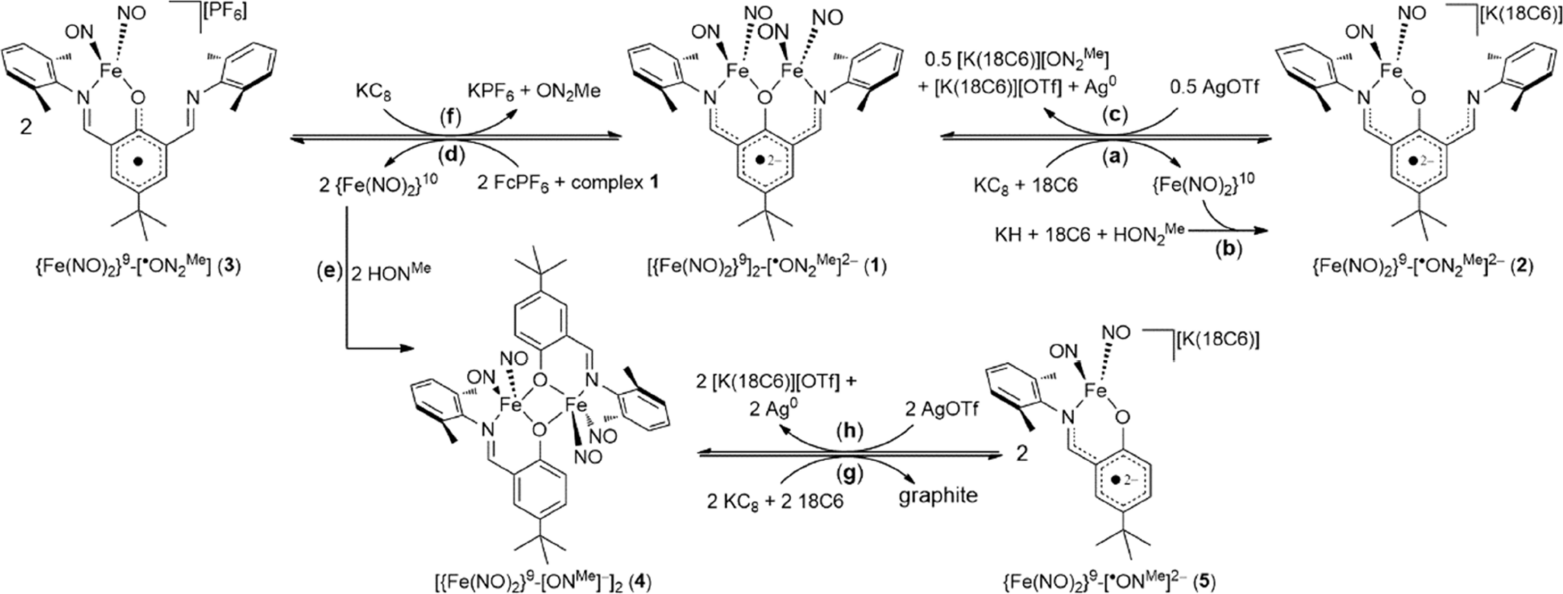
Redox Interconversion among Complexes **1–5** along with the Liberation/Transport of the [Fe(NO)_2_]^10^ Dinitrosyl Iron Unit (DNIU)

**Figure 1:**
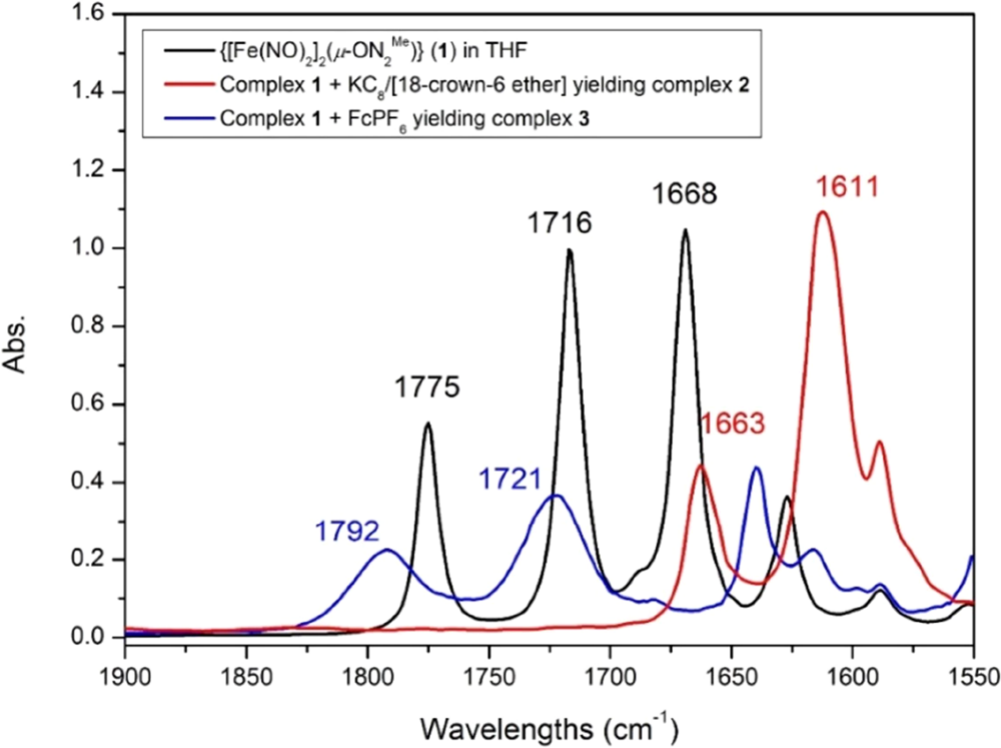
IR spectra (THF) for [Fe(NO)_2_(μ-ON_2_^Me^)Fe(NO)_2_] (**1**, black line) reacting with KC_8_+ [18-crown-6-ether] (**2**, red line) and reacting with FcPF_6_ (**3**, blue line), respectively.

**Figure 2:**
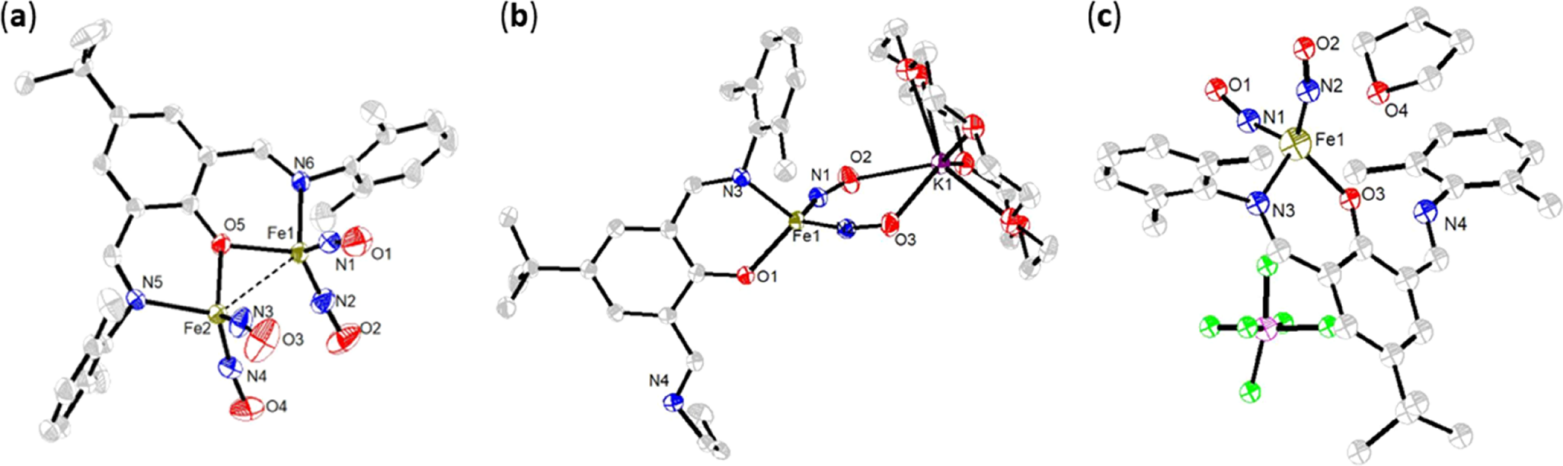
Crystallographic single-crystal X-ray structures of (a) [Fe(NO)_2_(μ-ON_2_^Me^)Fe(NO)_2_] (**1**), (b) [K-18-crown-6-ether)][(ON_2_^Me^)Fe(NO)_2_] (**2**), and (c) [(ON_2_^Me^)Fe(NO)_2_][PF_6_] (**3**). Selected bond lengths and bond angles are shown in Table S1.

As shown in Scheme 2 and Figure 1, the addition of 2 equiv of Fe(CO)_2_(NO)_2_ into the THF solution of 4-*tert*-butyl-2,6-bis(2,6-dimethylphenyliminomethyl)phenol (HON_2_^Me^) yielded dinuclear DNIC [(μ-ON_2_^Me^)Fe_2_(NO)_4_] (**1**). In contrast to the decoupled NO-vibrational peaks [IR ν_NO_ 1762 m, 1701 s, 1682 sh, 1650 s cm^–1^ (THF)] exhibited by electronically localized, singly *O*-alkoxide-bridged dinuclear {Fe(NO)_2_}^10^-{Fe(NO)_2_}^9^-[bdmap]^−^ DNIC [(NO)_2_Fe(μ-*O*-bdmap)Fe(NO)_2_(THF)] (bdmap = 1,3-bis(dimethylamino)-2-propanolate), the coupled NO-stretching frequencies [IR ν_NO_ 1775 s, 1716 vs, 1668 vs cm^–1^ (THF)] between two adjacent [Fe(NO)_2_] groups suggest the fully delocalized electronic structure of singly *O*-phenoxide-bridged dinuclear {Fe(NO)_2_}^9^-{Fe(NO)_2_}^9^-[^·^ON_2_^Me^]^2–^ DNIC **1**.^38^ Single-crystal X-ray diffraction of complex **1** is depicted in Figure 2a, and the selected bond distances and angles are presented in ss. The Fe–N(O) bond lengths of 1.663(3)/1.674(3) Å (mean value 1.6675(3) Å) and the N–O bond lengths of 1.179(3)/1.190(3) Å (mean value 1.1845(3) Å) are within the range of the published {Fe(NO)_2_}^10^-{Fe(NO)_2_}^9^ DNICs.^29,34,36^ The shorter Fe(1)···Fe(2) distance of 2.7394(6) Å found in dinuclear DNIC **1**, comparable to that of electronically delocalized {Fe(NO)_2_}^10^-{Fe(NO)_2_}^9^ dinuclear DNIC [Fe(NO)_2_(μ-bdmap)Fe(NO)_2_] (Fe(1)···Fe(2) distance of 2.8063(6) Å),^39^ rationalizes that the structurally rigid [ON_2_^Me^] skeleton tightly constrains two [Fe(NO)_2_] cores, resulting in the partial Fe···Fe interaction. Furthermore, the slightly shorter O–C_phenolate_ bond length of 1.313 Å, compared to the reported phenolate-bridged dinuclear DNIC [Fe(NO)_2_(μ-OPh)Fe(NO)_2_]^2–^ (1.338 Å) and phenolate-bound DNIC [Fe(NO)_2_(OPh)_2_]^−^ (1.330 Å),^31,34,74^ may suggest electron delocalization among two [Fe(NO)_2_] units and the supporting skeleton, rather than purely electron shuttling between two iron centers.

**Figure 3:**
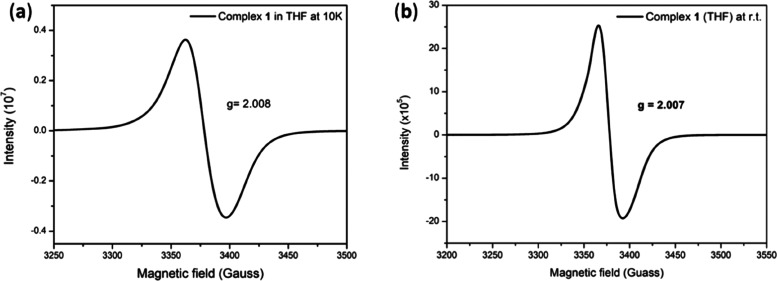
X-band EPR spectra (THF) of (a) [Fe(NO)_2_(μ-ON_2_^Me^)Fe(NO)_2_] (**1**) (10.4 mM) with *g*_av_ = 2.007 at 10 K and (b) with *g*_av_ = 2.008 at room temperature (9.484191 GHz, power = 15 mW, receiver gain = 20 for 10 K and 5 for room temperature).

As displayed in Figures 3 and S1, EPR spectra present an isotropic signal at *g* = 2.008 (THF) at 10 K and *g* = 2.007 (THF)/*g* = 2.008 (solid) at room temperature. Synchronized with the coupled ν_NO_ frequencies, EPR analysis of DNIC **1** implicates that an unpaired-electron delocalization among [ON_2_^Me^]^−^ skeleton and two [Fe(NO)_2_] cores results in the [{Fe(NO)_2_}^9^-{Fe(NO)_2_}^9^-[^·^ON_2_^Me^]^2–^] electronic structure with free-radical featured doublet ground state (Figure S20). This assignment is also supported by the Fe K-edge pre-edge feature (Figure S2), where the pre-edge energy of DNIC **1** (7113.6 eV) is slightly higher than those (7113.4–7113.5 eV) of the published electronically localized {Fe(NO)_2_}^10^-{Fe(NO)_2_}^9^ DNICs.^29,34,36^ With spin-only {Fe(NO)_2_}^9^ [Fe(NO)_2_(SPh)_2_]^−^ (*S* = 1/2) as a spin reference, the spin quantification of complex **1** in THF implies the spin population of *S* = 1/2 as 93.78% (Figure S3). Also, the effective magnetic moment of 1.71 μ_B_ revealed by solution magnetic measurement (Evans method) indicates that an unpaired electron delocalizes among two adjacent [Fe(NO)_2_] cores and its redox-accessible ligand (Figure S4). In comparison with the electronically localized, singly *O*-alkoxide-bridged {Fe(NO)_2_}^10^-{Fe(NO)_2_}^9^-[*O*-bdmap]^−^ dinuclear DNIC [Fe(NO)_2_(μ-bdmap)Fe(NO)_2_(THF)] showing an isotropic EPR signal (*g*_DNIC_ = 2.016 (five-coordinate {Fe(NO)_2_}^9^ motif in THF)) at 77 K,^38^ it is presumed that the “constrained” and “hard” *O*-phenoxide-bridged [ON_2_^Me^]^−^ ligand not only polarizes two irons to preserve the {Fe(NO)_2_}^9^ cores but also acts as an electron reservoir to promote/stabilize electronically delocalized dinuclear DNIC [{Fe(NO)_2_}^9^-{Fe(NO)_2_}^9^-[^·^ON_2_^Me^]^2–^] (**1**). That is, for the redox-active [ON_2_^Me^]^−^-coordinated DNIC **1**, the [{Fe(NO)_2_}^9^-{Fe(NO)_2_}^9^-[^·^ON_2_^Me^]^2–^] electron configuration may be suggested.

### 2.2 Redox Interconversion between DNIC **1** and DNICs 2/3 along with Liberation of the [Fe(NO)_2_]^10^ Unit

**Scheme 3:**
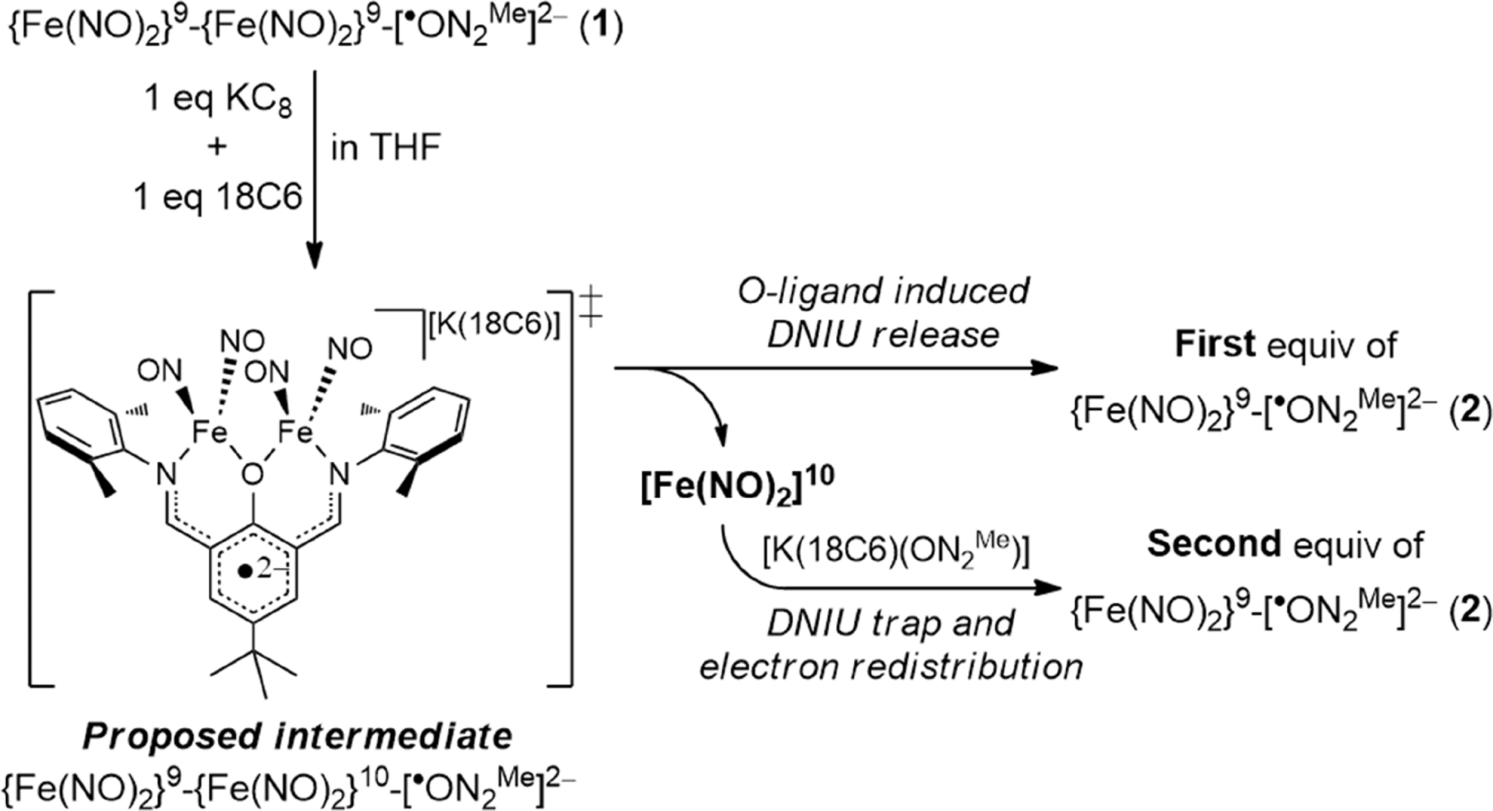
Proposed Mechanism for the Reduction of DNIC **1** to DNIC **2** along with [Fe(NO)_2_]^10^ Liberation

**Figure 4:**
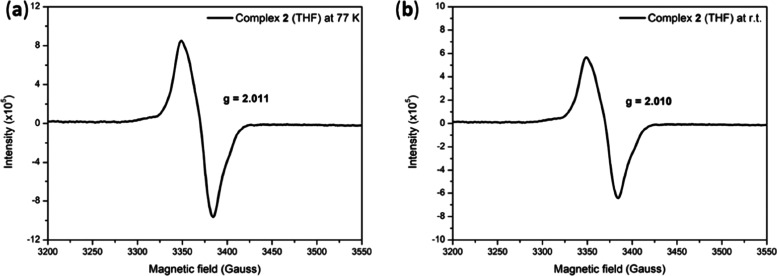
X-band EPR spectra (THF) of (a) [K(18-crown-6 ether)][(ON_2_^Me^)Fe(NO)_2_] (**2**) (4.0 mM) with *g*_av_ = 2.011 at 77 K and (b) with *g*_av_ = 2.010 at room temperature (9.484191 GHz, power = 15 mW, receiver gain = 30).

In order to further investigate the redox properties of dinuclear DNIC **1**, reactions of DNIC **1** with KC_8_ and AgOTf, respectively, were conducted. The addition of DNIC **1** into the THF solution of 1 equiv of KC_8_ afforded the brownish red solution along with releasing NO_(g)_ and N_2_O_(g)_ (headspace detected by GC, Figure S5). As displayed in Scheme 2a and Figure 1, IR ν_NO_ of the brownish red solution appeared at 1663 s, 1611 vs cm^–1^ (THF) suggesting the generation of mononuclear DNIC [K-18-crown-6-ether)][(ON_2_^Me^)Fe(NO)_2_] (**2**) characterized by a single-crystal X-ray structure (Figure 3b and Table S1). In comparison with the reported {Fe(NO)_2_}^9^ DNICs (1.160–1.186 Å), the longer N–O bond lengths of 1.217(2)/1.223(3) Å (mean value 1.220(2) Å) in DNIC **2** may be ascribed to the interaction between N–O···K-18-crown-6-ether (Figure 2b and Table S1).^34^ Similar to the O–C_phenolate_ bond length of 1.313 Å in electronically delocalized dinuclear DNIC **1**, the significant shortened O–C_phenolate_ of 1.299(3) Å in DNIC **2** suggests electron delocalization between the [Fe(NO)_2_] unit and supporting ligand via the polarization of Fe center by the hard *O*-phenoxide group. Compared to the reported {Fe(NO)_2_}^10^ DNICs (7113.0–7113.4 eV) and {Fe(NO)_2_}^9^ DNICs (7113.5–7114.2 eV), the higher Fe K-edge pre-edge energy (7113.6 eV) displayed by complex **2** explicates that the electron delocalization between the [Fe(NO)_2_]^10^ dinitrosyl iron unit (DNIU) and [ON_2_^Me^]^−^ ligand framework leads to [{Fe(NO)_2_}^9^-[^·^ON_2_^Me^]^2–^] electronic structure (Figure S2). The proposed electronic structure is verified by EPR analysis. The solid-state DNIC **2** displays a rhombic signal at *g*_av_ = 2.018 (*g*_1_= 2.033, *g*_2_ = 2.016, *g*_3_ = 2.007) at ambient temperature. THF solution of DNIC **2** presents signals with *g*_av_ = 2.011 at 77 K and *g*_av_ = 2.010 at room temperature, indicative of the moderate exchange coupling of the [^·^N_2_^Me^]^2–^ ligand radical and {Fe(NO)_2_}^9^ motif (Figures 4 and S6). As opposed to iron-based reduction of the fully delocalized mixed-valence {Fe(NO)_2_}^10^-{Fe(NO)_2_}^9^ [(μ-SEt)Fe(NO)_2_]_2_^–^ yielding dinuclear {Fe(NO)_2_}^10^-{Fe(NO)_2_}^10^ DNIC [(μ-SEt)Fe(NO)_2_]_2_^2–^, reduction of DNIC **1** with 1 equiv of KC_8_ produced DNIC **2** along with release of the proposed {Fe(NO)_2_}^10^ motif. Owing to the proposed extrusion of 1 equiv of the {Fe(NO)_2_}^10^ unit leading to the liberation of NO/N_2_O in the reduction process, in the control experiment, 1 equiv of KON_2_^Me^ (in situ preparation from KH, HON_2_^Me^ and 18-crown-6-ether) was added into the reaction mixture (DNIC **1** + [KC_8_ + 18-crown-6-ether]) to capture the released {Fe(NO)_2_}^10^ unit. As shown in Scheme 2a,b and Figure S7, the reaction of DNIC **1** with 1 equiv of KC_8_ and KON_2_^Me^ yielded 2-fold DNIC **2** demonstrated by UV–vis and IR spectroscopies. Presumably, one-electron reduction of DNIC **1** leads to the formation of the proposed intermediate {Fe(NO)_2_}^10^-{Fe(NO)_2_}^9^-[^·^ON_2_^Me^]^2–^ DNIC (not observed). Subsequently, the “constrained” and “hard” π-conjugated *O*-phenoxide [ON_2_^Me^]^−^ ligand preserves the {Fe(NO)_2_}^9^ core, leading to the formation of the first equivalent of DNIC **2** and 1 equiv of a [Fe(NO)_2_]^10^ unit. The latter can then be trapped with 1 equiv of free ligand KON_2_^Me^, generating a second equiv of DNIC **2** (Scheme 3). Reversibly, the oxidation of DNIC **2** with 0.5 equiv of AgOTf produced 0.5 equiv of DNIC **1** (Figure S8). This may be rationalized by the recombination of DNIC **2** and the thermodynamically unstable one-electron-oxidized {Fe(NO)_2_}^9^-[ON_2_^Me^]^−^ species, giving rise to DNIC **1** along with the release of the [ON_2_Me]^−^ ligand (Scheme 2c).

**Figure 5:**
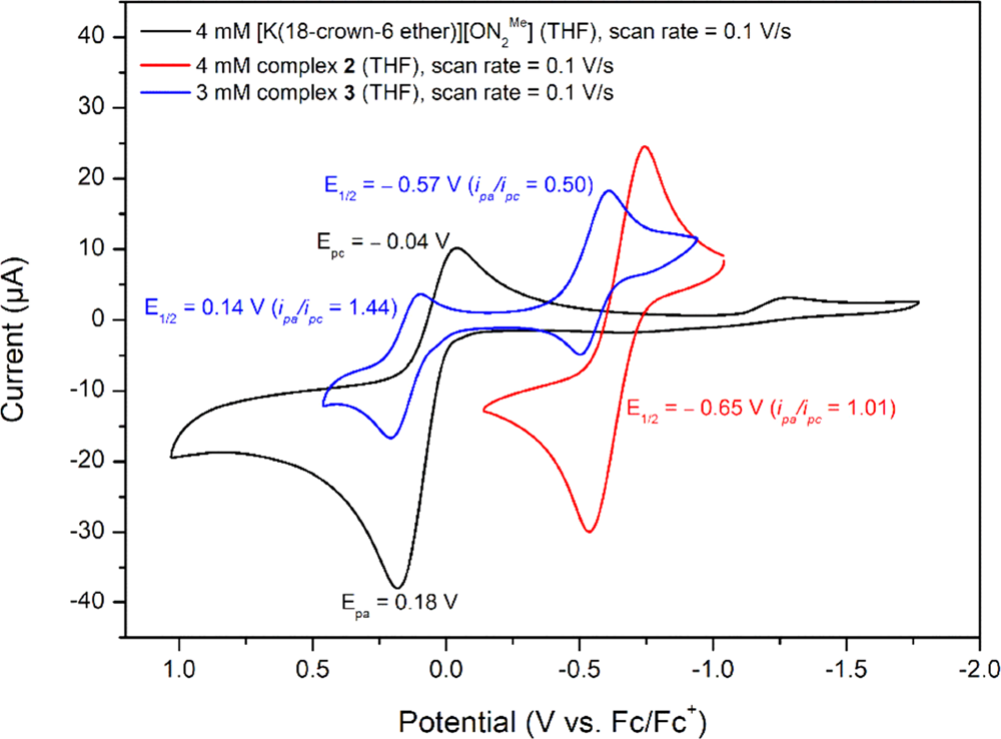
Cyclic voltammograms of 4 mM [K(18-crown-6-ether)][ON_2_^Me^] (deprotonated [ON_2_^Me^] ligand), 4 mM complex **2,** and 3 mM complex **3** measured in a THF solution with 0.2 M [*n*-Bu_4_N][PF_6_] as the supporting electrolyte (ferrocene as the internal standard at room temperature, scan rate 0.1 V/s).

Accompanied by NO_(g)_ and N_2_O_(g)_ detected by GC (Figure S9), the oxidation of DNIC **1** with 1 equiv of [Cp_2_Fe][PF_6_] in THF generated the brown solution with IR ν_NO_ 1792 s, 1721 vs cm^–1^ (THF), implicating the formation of {Fe(NO)_2_}^9^ DNIC [(ON_2_^Me^)Fe(NO)_2_][PF_6_] (**3**) characterized by single-crystal X-ray diffraction (Scheme 2d, Figures 1, 3c and Table S1). The Fe–N(O) bond lengths of 1.674(6)/1.683(6) Å (mean value 1.6785(6) Å) and the N–O bond lengths of 1.177(7)/1.151(7) Å (mean value 1.164(7) Å) in DNIC **3** fall in the range of 1.661–1.696 and 1.160–1.186 Å, respectively, observed for {Fe(NO)_2_}^9^ DNICs.^29,33,36,39,73^ Additionally, the O–C_phenol_ bond length of 1.300(6) Å, compared to the published O–C_phenoxyl radical_ bond lengths (1.250–1.300 Å), confirms the ligand-based oxidation process and the {Fe(NO)_2_}^9^-[^·^ON_2_^Me^] electronic structure in DNIC **3**.^75−77^ Oxidation of DNIC **1** with 1 equiv of [Cp_2_Fe][PF_6_] in THF yielding complex **3** and NO_(g)_/N_2_O_(g)_ may suggest the release of the [Fe(NO)_2_] unit. Presumably, the one-electron ligand-based oxidation causes electron redistribution of the transient singly *O*-phenoxide-bridged {Fe(NO)_2_}^9^-{Fe(NO)_2_}^9^-[ON_2_^Me^]^−^ [Fe(NO)_2_(μ-ON_2_^Me^)Fe(NO)_2_]^+^ intermediate, resulting in the release of [Fe(NO)_2_] unit and the preservation of {Fe(NO)_2_}^9^ core by the π-conjugated *O*-phenoxyl [^·^ON_2_^Me^] ligand framework. This is contrary to the published [Fe_2_(^Pyrr^PDI)(NO)_4_]^0/+^ DNIC system, where free NO gas is not observed. The one-electron oxidative transformation of [{Fe(NO)_2_}_2_]^10/10^ [Fe_2_(^Pyrr^PDI)(NO)_4_] DNIC to [{Fe(NO)_2_}_2_]^9/10^ [Fe_2_(^Pyrr^PDI)(NO)_4_]^+^ DNIC triggers the site lability of electron-deficient {Fe(NO)_2_}^10^ motif to undergo intra/intermolecular N–N bond formation, leading to the formation of {Fe(NO)_2_}^9^ DNIC [Fe(^Pyrr^PDI)(NO)_2_]^+^ along with N_2_O_(g)_ and Fe_*x*_O_*y*_^78^ In order to capture the extruded [Fe(NO)_2_] motif, 1 equiv of 4-*tert*-butyl-2-(2,6-dimethylphenyliminomethyl)phenol (HON^Me^) was added into the reaction mixture of DNIC **1** and [Cp_2_Fe][PF_6_]. As shown in Scheme 2d,e, DNIC **1** is oxidatively converted into DNIC **3** along with the formation of ON^Me^-bridged dinuclear {Fe(NO)_2_}^9^-{Fe(NO)_2_}^9^ DNIC [(μ-ON^Me^)_2_Fe_2_(NO)_4_] (**4**) characterized by IR ν_NO_ 1792 s, 1780 s, 1721 s, 1717 vs cm^–1^ (THF) and single-crystal X-ray structures (Figures S10 and S11). Obviously, the oxidation in [^·^ON_2_^Me^]^2–^-bridging ligand triggers the exclusion of {Fe(NO)_2_}^10^ motif and then produces mononuclear {Fe(NO)_2_}^9^-[^·^ON_2_^Me^] DNIC **3** and dinuclear {Fe(NO)_2_}^9^-{Fe(NO)_2_}^9^ DNIC **4**. Commensurate with ligand-based oxidation of {Fe(NO)_2_}^9^ DNIC [Fe(NO)_2_(N(Mes)(TMS))_2_]^−^ yielding {Fe(NO)_2_}^9^/[^·^N(Mes)(TMS)]-coupled DNIC [Fe(NO)_2_(N(Mes)(TMS))(^·^N(Mes)(TMS))],^32^ both the isotropic EPR signal with *g* = 2.038 and Fe K-edge pre-edge energy at 7114.0 eV rationalize the electron-deficient {Fe(NO)_2_}^9^-[^·^ON_2_^Me^] electronic structure of DNIC **3** that is stabilized by the weak coordination of THF molecule with the Fe–O_THF_ distance as 2.728^2^ Å (Figures 2c, S2, and S12). The chemical reduction of DNIC **3** to DNIC **1** and DNIC **2** was observed when the THF solution of DNIC **3** was treated with 1 and 2 equiv of KC_8_, respectively (Figure S13 and Scheme 2a,f). Cyclic voltammograms (CV) of DNICs **2** and **3** were investigated in THF (3–4 mM) with 0.2 M [*n*-Bu_4_N][PF_6_] as a supporting electrolyte. As shown in Figures 5 and S14, DNIC **3** displays two quasi-reversible redox couples. The first redox couple at *E*_1/2_ = 0.14 V (*E*_pa_ = 0.19 V, *E*_pc_ = 0.09 V, Δ*E*_p_ = 0.10 V, *i*_pa_/*i*_pc_ = 1.44 vs Fc/Fc^+^) in DNIC **3**, corresponding to the redox behavior of deprotonated [ON_2_^Me^]^−^ ligand at *E*_pa_ = 0.18 V and *E*_pc_ = −0.04 V (vs Fc/Fc^+^), suggests the conversion of {Fe(NO)_2_}^9^-[^·^ON_2_^Me^] DNIC **3** into {Fe(NO)_2_}^9^-[ON_2_^Me^]^−^ transient intermediate. The second redox couple of DNIC **3** at *E*_1/2_ = −0.57 V (*E*_pc_ = −0.62 V, *E*_pa_ = −0.52 V, Δ*E*_p_ = 0.10 V, *i*_pa_/*i*_pc_ = 0.50 vs Fc/Fc^+^), similar to the anodic scan of DNIC **2** at *E*_1/2_ of −0.65 V (*E*_pc_ = −0.75 V, *E*_pa_ = −0.55 V, Δ*E*_p_ = 0.10 V, *i*_pa_/*i*_pc_ = 1.01 vs Fc/Fc^+^ (Figures 5 and S15), implicates the transformation of the {Fe(NO)_2_}^9^-[ON_2_^Me^]^−^ transient intermediate into {Fe(NO)_2_}^9^-[^·^ON_2_^Me^]^2–^ DNIC **2**. The successive electrochemical shuttle among {Fe(NO)_2_}^9^-[^·^ON_2_^Me^] DNIC **3**, {Fe(NO)_2_}^9^-[ON_2_^Me^]^−^ transient intermediate, and {Fe(NO)_2_}^9^-[^·^ON_2_^Me^]^2–^ DNIC **2** is kinetically accessible. However, isolating the {Fe(NO)_2_}^9^-[ON_2_^Me^]^−^ transient intermediate is a challenge. The successful isolation of ON^Me^-bridged dinuclear {Fe(NO)_2_}^9^-{Fe(NO)_2_}^9^ DNIC **4** [(μ-ON^Me^)_2_Fe_2_(NO)_4_] (Scheme 2d,e and Figure S16) indicates that the [(ON_2_^Me^)Fe(NO)_2_] transient intermediate with the {Fe(NO)_2_}^9^-[ON_2_^Me^]^−^ electronic configuration is thermodynamically unstable owing to the dimerization of [(ON_2_^Me^)Fe(NO)_2_] intermediates to form {Fe(NO)_2_}^9^-{Fe(NO)_2_}^9^ DNIC [(μ-ON_2_^Me^)_2_Fe_2_(NO)_4_] being sterically inaccessible. In addition, the electrochemical reversibility among {Fe(NO)_2_}^9^-[^·^ON_2_^Me^]^2–^ (**2**) ↔ {Fe(NO)_2_}^9^-[ON_2_^Me^]^−^ ↔ {Fe(NO)_2_}^9^-[^·^ON_2_^Me^] (**3**) electronic states demonstrates that by relying on the “constrained” and “hard” π-conjugated *O*-phenoxide [ON_2_^Me^]^−^ ligand framework, DNIC **1** could serve as [Fe(NO)_2_]^10^ unit storage and transport, triggered by redox reaction of complex **1**.

**Figure 6:**
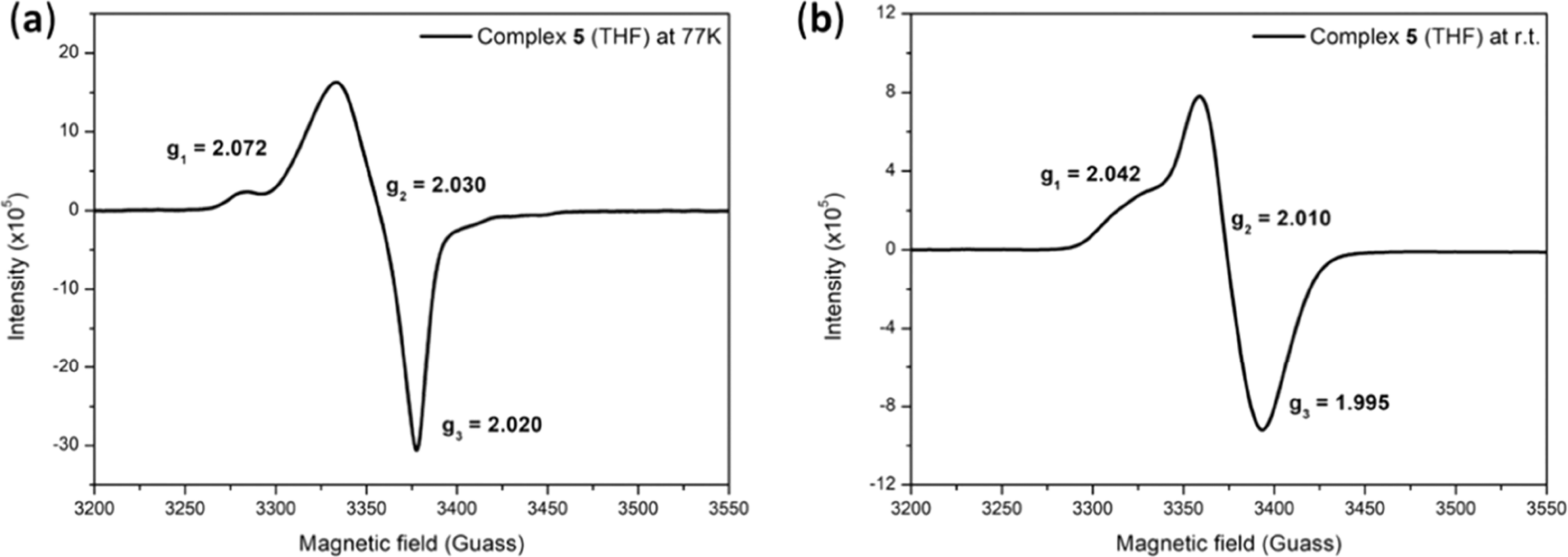
X-band EPR spectra (THF) of (a) [K-18-crown-6-ether][(ON^Me^)Fe(NO)_2_] (**5**) (4.8 mM) with *g*_1_ = 2.072, *g*_2_ = 2.030, and *g*_3_ = 2.020 at 77 K and (b) with *g*_1_ = 2.042, *g*_1_ = 2.010, and *g*_1_ = 1.995 at room temperature (9.484191 GHz, power = 15 mW, receiver gain = 30).

As shown in Figure S11 and Table S1, the bond lengths of Fe–N(O) of 1.711(2)/1.730(2) Å (mean value 1.7205(2) Å) and the N–O bond lengths of 1.168(2)/1.189(5) Å (mean value 1.178(2) Å) in DNIC **4** are comparable to the reported dinuclear {Fe(NO)_2_}^9^-{Fe(NO)_2_}^9^ DNIC [Fe(NO)_2_(μ-bdmap)]_2_.^38^ The O–C_phenolate_ bond length of 1.333(2) Å in DNIC **4** is consistent with of phenoxide-bound {Fe(NO)_2_}^9^ and {Fe(NO)_2_}^10^ DNICs (1.330–1.338) Å.^31,34,74^ Noticeably, the longer Fe–O_phenoxide_ bond length of 2.076(3) Å, compared with the reported Fe–O_phenoxide_ bond length (1.884–2.018 Å) of {Fe(NO)_2_}^9^ DNICs, explicates weak dipolar coupling between two adjacent {Fe(NO)_2_}^9^ cores.^31,34^ The weak dipolar coupling makes DNIC **4** show a rhombic EPR signal at *g*_1_ = 2.066, *g*_2_ = 2.015, *g*_3_ = 2.004 (*g*_av_ = 2.028) at 77 K, and an isotropic EPR signal at *g* = 2.038 at 298 K (Figure S17). In contrast to one-electron chemical reduction of {Fe(NO)_2_}^9^ [(EtS)_2_Fe(NO)_2_]^−^ and [(MeO)_2_Fe(NO)_2_]^−^ yielding the diamagnetic {Fe(NO)_2_}^10^ [(EtS)_2_Fe(NO)_2_]^2–^ and {Fe(NO)_2_}^10^-{Fe(NO)_2_}^10^ [(μ-MeO)Fe(NO)_2_]_2_^2–^, respectively,^34^ the cleavage of bridged *O*-phenoxide triggered by the reduction of DNIC **4** with 1 equiv of KC_8_ generates the mononuclear [K-18-crown-6-ether][(ON^Me^)Fe(NO)_2_] (**5**) characterized by IR ν_NO_ 1658 s, 1605 vs cm^–1^ (THF) and the single-crystal X-ray structure (Figures S16 and S18 and Table S1). The Fe–N(O) bond lengths of 1.649(5)/1.650(5) Å (mean value 1.6495(5) Å), the N–O bond lengths of 1.199(5)/1.194(5) Å (mean value 1.1965(5) Å), and the O–C_phenolate_ bond length of 1.299(3) Å in DNIC **5** are consistent with those of [K-18-crown-6-ether)][(ON_2_^Me^)Fe(NO)_2_] (**2**). Interestingly, in addition to Fe K-edge pre-edge energy (7113.7 eV) of DNIC **5** being close to that (7113.6 eV) of DNIC **2**, it is noticed that EPR behavior is similar to that of DNIC **2** (Figures 6, S2, and S19). The solid-state EPR spectra of DNIC **5** present a rhombic EPR signal at *g*_av_ = 2.018 (*g*_1_ = 2.033, *g*_2_ = 2.016, and *g*_3_ = 2.007), while the THF solution of DNIC **5** displays rhombic EPR signals located at *g*_av_ = 2.039 (*g*_1_ = 2.072, *g*_1_, and *g*_1_ = 2.020) at 77 K and *g*_av_ = 2.015 (*g*_1_ = 2.042, *g*_2_ = 2.010, and *g*_3_ = 1.995) at ambient temperature. The combination of the EPR study and Fe K-edge pre-edge result suggests that the electronic structure of DNIC **5** may be best described as [{Fe(NO)_2_}^9^-[^·^ON^Me^]^2–^]. Further insights into the electronic structure of DNICs **2** and **5** will be delineated in terms of magnetic measurements.

### 2.3 Magnetic Measurements toward Electron Configuration of DNICs **1–5**

The magnetic susceptibility of microcrystalline samples of DNICs **1**–**5** was measured in the temperature range of 2.00–300 K under an applied field of 5000 G. The molar magnetic susceptibility (χ_m_) of DNIC **1**, as shown in Figure S20, increases from 2.25 × 10^–3^ cm^3^/mol at 300 K to 0.236 cm^3^/mol at 2 K, while the corresponding χ_m_*T* value decreases from 0.674 cm^3^ K/mol at 300 K to 0.472 cm^3^ K/mol at 2 K. Since FT-IR, EPR study, and Fe K-edge pre-edge energy unveil the electronic structure of DNIC **1** as [{Fe(NO)_2_}^9^-{Fe(NO)_2_}^9^-[^·^ON_2_^Me^]^2–^] with the free-radical featured doublet ground state, the cyclic three-spin/two-*J*-value system is considered as a geometrically spin-frustrated system with a C_2_-symmetric isosceles structure (Figure S20a). Furthermore, based on the sharp χ_m_ increment below Neel temperature deducing the presence of magnetic long-range ordering (*T*_N_ = 9.06 K) in the χ_m_ vs *T* plot, the frustration parameter *f* = |θ|/*T*_N_ = 5.19 verifies that the spin–spin interaction of complex **1** could be recognized as spin frustration (Figure S20b). The isotropic exchange is described by spin Hamiltonian (*Ĥ* = −*J*_1_*Ŝ*_1_*Ŝ*_3_ – *J*_1_*Ŝ*_2_*Ŝ*_3_ – *J*_2_*Ŝ*_1_*Ŝ*_2_) for a triangle of spin doublets, where *J*_1_ and *J*_2_ are the exchange parameters of [{Fe(NO)_2_}^9^-[^·^ON_2_^Me^]^2–^] and [{Fe(NO)_2_}^9^-{Fe(NO)_2_}^9^] electronic structures, respectively. With the spin-frustrated framework of the isotropic Heisenberg–Dirac–van Vleck (HDVV) model (Figure S20c,d), the exchange parameters cannot be determined unequivocally, and the energy pattern for the isosceles triangle (C_2_) includes the low-lying ground spin doublet (^2^B_2_), an excited spin doublet (^2^A_1_) at δ energy (δ = *J*_1_ – *J*_2_), and an excited spin quartet (^4^A_2_) at (3*J*_av_ + δ)/2 energy (*J*_av_ = (2*J*_1_ + *J*_2_)/3). The fitting of the magnetic data for an isosceles spin frustrated situation could be expressed by eq 1.^79−83^
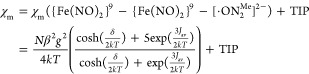
1

The best fit (*R*^2^ = 0.9991) to the χ_m_ vs temperature (2–300 K) curve with a fixed EPR *g* value (*g* = 2.008) gives *J*_av_ = −146.67(24) and δ = 16.51(13) cm^–1^ with the temperature-independent paramagnetism (TIP) held constant at 18.75(19) × 10^–6^ cm^3^/mol (Figure S20c). The SQUID result indicates that the competing spin interaction among [^·^ON_2_^Me^]^2–^ and the adjacent two {Fe(NO)_2_}^9^ cores in spin-frustrated DNIC **1** contributes to the C_2_-induced mixing of (0, 1/2) and (1, 1/2) magnetic states, resulting in ^2^A_1_ and ^2^B_2_ doublet states with a g value of 2.008 in EPR spectra (Figures 3, S1, and S20d).

With respect to DNIC **2** and DNIC **5** displaying a similar EPR pattern and Fe K-edge pre-edge energy, SQUID measurements were conducted to investigate their magnetic behavior. In comparison with Fe K-edge pre-edge energy (7113.0–7113.4 eV) of the reported {Fe(NO)_2_}^10^ DNICs, the higher pre-edge energy (DNIC **2**, 7113.6 eV; DNIC **5**, 7113.7 eV) suggests that the electronic structure of DNICs **2** and **5** is best described as [{Fe(NO)_2_}^9^-[^·^L]^2–^] ([^·^L]^2–^ = [^·^ON_2_^Me^]^2–^ for DNIC **2** and [^·^ON^Me^]^2–^ for DNIC **5**. The temperature-dependent magnetic susceptibility (χ_m_*T*) of DNIC **2** (decrease from 0.7996 cm^3^ K/mol at 300 K to 0.0365 cm^3^ K/mol at 2 K) and DNIC **5** (decrease from 0.7072 cm^3^ K/mol at 300 K to 0.1209 cm^3^ K/mol at 2 K) implicates the antiferromagnetic coupling between the {Fe(NO)_2_}^9^ core and ligand-based radical ([^·^ON_2_^Me^]^2–^ for DNIC **2** and [^·^ON^Me^]^2–^ for DNIC **5**). The equation χ_m_ = (2*Ng*^2^β^2^/*kT*)/[3 + exp(−*J*/*kT*)] derived from Heisenberg Hamiltonian (*Ĥ* = −*JŜ*_1_*Ŝ*_2_) is used to describe isotropic exchange between ligand radical and {Fe(NO)_2_}^9^ motif, leading to the singlet ground state (Figures S21a and S22a). In order to get the best fit of SQUID data, the Weiss constant (θ) was introduced to address the presence of intermolecular magnetic interaction at temperatures below 10 K. As shown in Figures S21b and S22b, the best fit of experimental data indicates that the ligand radical ([^·^L]^2–^) is weakly antiferromagnetic coupled with the [{Fe(NO)_2_}^9^] motif [DNIC **2**, *g* = 2.010 (fixed), *J* = −19.72(68) cm^–1^, θ = −6.70(2) K, TIP = 298.64(31) × 10^–6^ cm^3^/mol with *R*^2^ = 0.9913; DNIC **5**, *g* = 2.010 (fixed), *J* = −23.41(29) cm^–1^, θ = −5.44(9) K, TIP = 11.52(17) × 10^–6^ cm^3^/mol with *R*^2^ = 0.9998], leading to the magnetic ground state with total spin *S* = 0. Additionally, anisotropic EPR signals at 77 and 298 K (Figures 4, 5, S6, and S19) may originate from the significant population of the thermal-accessible triplet excited state in DNIC **2** and DNIC **5**.

In consideration of Fe K-edge pre-edge energy located at 7114.0 eV, the temperature-dependent magnetic susceptibility (χ_m_*T*) of DNIC **3**, decreasing from 0.8476 cm^3^ K/mol at 300 K to 0.2108 cm^3^ K/mol at 2 K, could be attributed to the weak antiferromagnetic coupling between {Fe(NO)_2_}^9^ motif and neutral [^·^ON_2_^Me^] phenoxyl radical (Figure S23). The best fitting [*g* = 2.038 (fixed), TIP = 88.97(55) × 10^–6^ cm^3^/mol with *R*^2^ = 0.9972] of magnetic data based on Heisenberg Hamiltonian (*Ĥ* = −*JŜ*_1_*Ŝ*_2_) explicates that EPR signals at 77 and 298 K originate from the thermal-accessible excited triplet state (*J* = −2.97(4) cm^–1^). For dinuclear {Fe(NO)_2_}^9^-{Fe(NO)_2_}^9^ DNIC **4**, the magnetic susceptibility (χ_m_*T*) is temperature-dependent (decrease from 0.9179 cm^3^ K/mol at 300 K to 0.4792 cm^3^ K/mol at 2 K) and could be fitted with Heisenberg Hamiltonian (*Ĥ* = −*JŜ*_1_*Ŝ*_2_) to give *g* = 2.038 (fixed), *J* = −1.76(8) cm^–1^ and TIP = 0.48(11) × 10^–6^ cm^3^/mol with *R*^2^ = 0.9912 (Figure S24). It is presumed that the longer Fe–O_phenoxide_ bond distance (2.076(3) Å) causes the weak antiferromagnetic coupling of the adjacent {Fe(NO)_2_}^9^ cores.

## 3 Conclusions

In this article, we present how the nature of “hard” and “constrained” π-conjugated *O*-phenoxide [ON_*n*_^Me^]^−^ (*n* = 1 or 2) ligands modulates redox interconversion via regulating the electronic structure of [Fe(NO)_2_] unit(s). The impact of this nature on electronically delocalized dinuclear DNIC **1** results in an isosceles spin-frustrated [{Fe(NO)_2_}^9^-{Fe(NO)_2_}^9^-[^·^ON_2_^Me^]^2–^] electronic structure, as evidenced by Fe K-edge pre-edge XAS (7113.6 eV), magnetic measurement (*J*_av_ = −146.67(24), δ = 16.51(13) cm^–1^), and isotropic EPR signals at *g* = 2.008. Accompanied by mononuclear DNIC **2** formation, the liberation of one equiv of [Fe(NO)_2_]^10^ unit triggered by one-electron reduction of dinuclear DNIC **1** is attributed to the preservation of the {Fe(NO)_2_}^9^ core by the “hard” and “constrained” π-conjugated *O*-phenoxide [ON_2_^Me^]^−^ ligand scaffold. In the case of one-electron oxidation of DNIC **1**, both mononuclear DNIC **3** and dinuclear DNIC **4** are generated in the presence of one equiv of HON^Me^ (DNIU catcher). The CV study and successful isolation of [{Fe(NO)_2_}^9^-[ON^Me^]^−^]_2_ DNIC **4** suggest steric hindrance against the dimerization of [{Fe(NO)_2_}^9^-[ON_2_^Me^]^−^] species, leading to ligand-based oxidation to form [{Fe(NO)_2_}^9^-[^·^ON_2_^Me^]] DNIC **3**. DNIC **5** derived from one-electron reduction of DNIC **4** exhibits similar EPR and magnetic behaviors to DNIC **2**, reiterating the influence of the “hard” and “constrained” π-conjugated *O*-phenoxide [ON_*n*_^Me^]^−^ ligands on preferring [{Fe(NO)_2_}^9^-[^·^ON_*n*_^Me^]^2–^] electronic configurations corroborated by Fe K-edge pre-edge XAS, SQUID measurement, and two isotropic EPR signals. The study underscores the crucial role of the “hard” and “constrained” π-conjugated *O*-phenoxide [ON_n_^Me^]^−^ ligand in guiding redox interconversions (Scheme 2) among complexes **1**–**5**, while simultaneously preserving the {Fe(NO)_2_}^9^ core(s) and facilitating the storage/transport of [Fe(NO)_2_]^10^ DNIU.

## 4 Experimental Selection

Manipulations, reactions, and transfers were conducted under a pure nitrogen atmosphere according to Schlenk techniques or in a glovebox (N_2_ atmosphere). Solvents were purified and distilled under nitrogen by utilizing suitable reagents [acetonitrile from CaH_2_–P_2_O_5_; diethyl ether/*n*-hexane/tetrahydrofuran (THF) from sodium benzophenone] and stored in dried, N_2_-filled flasks over 4 Å molecular sieves. 4-*tert*-butyl-2,6-bis(2,6-dimethylphenyliminomethyl)phenol (HON_2_^Me^) and 4-*tert*-butyl-2-(2,6-dimethylphenyliminomethyl)phenol (HON^Me^) were synthesized by the literature procedures.^84^ The reagents ferrocenium hexafluorophosphate ([Cp_2_Fe][PF_6_]) (Sigma-Aldrich), bis(triphenylphosphoranylidene)ammonium chloride ([PPN][Cl]) (Fluka), and sliver triflate (AgOTf) were used as received. Precursor [Fe(CO)_2_(NO)_2_] was synthesized based on the previous reports.^85^ Infrared spectra (IR) were recorded on a PerkinElmer-Frontier with sealed solution cells (0.1 mm, KBr windows). UV–vis spectra were recorded on an Agilent 8453 spectrophotometer equipped with a UNICOKU liquid N_2_ cryostat. ^1^H NMR spectra were obtained on a Varian Unity-500 MHz spectrometer. Analyses of carbon, hydrogen, and nitrogen were carried out with a CHN analyzer (Elementar Vario EL III CHN-OS Rapid).

### 4.1 Safety Considerations

Dinitrosyl iron complexes have the potential to release toxic and/or odorless gases like NO/N_2_O. Therefore, all reactions and storage involving these complexes must be conducted within a well-functioning fume hood and a glovebox filled with nitrogen (N_2_). It is strongly advised to carry out these experiments during regular working hours to ensure that, in the event of exposure, an appropriate emergency response can be promptly initiated. Before pressurizing any vessels, including the glovebox, Schenk line, and other containers, it is crucial to inspect them for any cracks. Additionally, these vessels should be properly secured using clamps, weights, or other appropriate methods.

### 4.2 Preparation of [Fe(NO)_2_(μ-ON_2_^Me^)Fe(NO)_2_] (**1**)

A THF solution (10 mL) of [Fe(CO)_2_(NO)_2_] (0.5 mmol) prepared freshly was added to the 25 mL Schlenk flask containing 4-*tert*-butyl-2,6-bis(2,6-dimethylphenyliminomethyl)phenol (HON_2_^Me^) (1.03 mg, 0.25 mmol) by cannula under N_2_. The reaction mixture was allowed to stir for 2 days at ambient temperature. The resulting green solution was dried under vacuum, and *n*-hexane (30 mL) was then added to dissolve the residual. The mixture solution was filtered through Celite to remove the insoluble solid, and the brown filtrate was then dried under vacuum to obtain the dark-brown solid [Fe(NO)_2_(μ-ON_2_^Me^)Fe(NO)_2_] (**1**) (101 mg, 63%). The *n*-hexane solution of complex **1** was kept at −20 °C for 2 weeks, leading to dark green crystals suitable for X-ray crystallography. IR ν_NO_ 1775 s, 1716 vs, 1668 vs cm^–1^ (THF); 1779 s, 1717 vs, 1669 vs cm^–1^ (KBr). Absorption spectrum (THF) λ_max_/nm (ε*/*M^–1^ cm^–1^): 355 (8744), 425 sh (3670), 700 (1367), 800 (1000). Anal. calcd for C_28_H_31_Fe_2_N_6_O_5_: C, 52.28; H, 4.86; N,13.06. Found: C, 53.46; H, 5.24; N,12.74.

### 4.3 Preparation of [K-18-Crown-6-ether][(ON_2_^Me^)Fe(NO)_2_] (**2**)

Complex **1** (64 mg, 0.1 mmol), KC_8_ (14 mg, 0.1 mmol), and 18-crown-6-ether (26 mg, 0.1 mmol) were added to a Schlenk tube containing 1 equiv of KON_2_^Me^ (in situ preparation from KH, HON_2_^Me^, and 18-crown-6-ether in 3 mL of THF) under a N_2_ atmosphere at 0 °C and stirred for 2 h. Diethyl ether was added into the resulting reddish brown solution, and the precipitate was dried under vacuum to obtain the yellowish red solid [K-18-crown-6 ether)][(ON_2_^Me^)Fe(NO)_2_] (**2**) (isolated yield 72 mg (88.2%)). The THF solution of complex **2** was layered with diethyl ether (15 mL) at room temperature for 1 week to obtain dark-red crystals suitable for X-ray crystallography. IR ν_NO_ 1663 s, 1611 vs cm^–1^ (THF). Absorption spectrum (THF) [λ_max_, nm (ε, M^–1^ cm^–1^)]: 480 (4000). Anal. calcd for C_40_H_55_FeKN_4_O_9_: C, 57.83; H, 6.67; N, 6.74. Found: C, 57.63; H, 7.07; N, 6.23.

### 4.4 [Fe(NO)_2_]^10^ Unit Captured upon the Reduction of Complex **1** with KC_8_ + 18-Crown-6-ether

(A) Experiment: the mixture of complex **1** (64 mg, 0.1 mmol) and 1.0 equiv of KC_8_ (14 mg, 0.1 mmol) + 18-crown-6-ether (26 mg, 0.1 mmol) was added into the Schlenk tube containing 3 mL of THF under a N_2_ atmosphere at 0 °C. After the reaction solution was stirred for 1 h and subsequently stood for 30 min, the top layer solution was measured by IR and UV–vis (diluted five times prior to measurement), as shown in Figure S3. The IR ν_NO_ 1663 s, 1611 vs cm^–1^ (THF) and UV–vis spectra at 480 nm suggest the formation of 1 equiv of [Na-18-crown-6-ether)][Fe(NO)_2_(ON_2_^Me^)] (**2**). (B) Control experiment: the mixture of complex **1** (64 mg, 0.1 mmol), 1.0 equiv of KC_8_ (14 mg) + 18-crown-6-ether (52 mg), and 1.0 equiv of KON_2_^Me^ (14 mg) was added into the Schlenk tube containing 3 mL of THF under a N_2_ atmosphere at 0 °C. After the reaction solution was stirred for 1 h and subsequently stood for 30 min, the top layer solution was measured by IR and UV–vis (diluted five times prior to measurement), as shown in Figure S3. In contrast to the reaction shown (A) above, the released [Fe(NO)_2_]^10^ unit in the control experiment (B) was captured by the extra 1 equiv of KON_2_^Me^ yielding 2-fold DNIC **2** based on the IR and UV–vis spectra.

### 4.5 Oxidation of Complex [Na-18-Crown-6-ether)][(ON_2_^Me^)Fe(NO)_2_] (**2**)

THF solution (3 mL) of complex **2** (83 mg, 0.1 mmol) was added into a Schlenk tube containing 0.5 equiv of silver triflate (12.8 mg, 0.05 mmol) under a N_2_ atmosphere. The reaction solution was stirred for 1 h, and then the solution was gradually converted to green solution at 0 °C. The appearance of IR ν_NO_ stretching frequencies 1775 s, 1716 vs, 1668 vs cm^–1^ (THF) demonstrated the formation of the known complex **1**, as shown in Figure S4.

### 4.6 Preparation of [(ON_2_^Me^)Fe(NO)_2_][PF_6_] (**3**)

Complex **1** (64 mg, 0.1 mmol) and [Cp_2_Fe][PF_6_] (33 mg, 0.1 mmol) were added into a Schlenk tube containing 3 mL of THF under a N_2_ atmosphere at 0 °C, and the mixture solution was stirred for 1 h. The resulting dark brown solution was precipitated by diethyl ether, and the residue was dried under vacuum to obtain the brown solid [(ON_2_^Me^)Fe(NO)_2_][PF_6_] (**3**) (isolated yield 38 mg (57%)). The THF solution of complex **3** was layered with diethyl ether (15 mL) at room temperature for 1 week to obtain dark-red crystals suitable for X-ray crystallography. IR ν_NO_ 1792 s, 1721 vs cm^–1^ (THF). Absorption spectrum (THF) [λ_max_, nm (ε, M^–1^ cm^–1^)]: 480 (4000). Anal. calcd forC_28_H_31_F_6_FeN_4_O_3_P: C, 50.02; H, 4.65; N, 8.33. Found: C, 51.63; H, 5.28; N, 7.53.

### 4.7 [Fe(NO)_2_]^10^ Unit Captured upon Oxidation of Complex **1** with [Cp_2_Fe][PF_6_]

(A) Blank experiment: complex **1** (64 mg, 0.1 mmol) and 1.0 equiv of HON^Me^ (28 mg, 0.1 mmol) were added into the Schlenk tube containing 3 mL of THF under a N_2_ atmosphere at room temperature. After the reaction solution was stirred for 1 h and, subsequently, stood for 30 min, the top layer THF solution was detected by IR, as shown in Figures 1 and S6. IR ν_NO_ 1775 s, 1716 vs, 1668 vs cm^–1^ (THF) shows no reaction. (B) Control experiment: complex **1** (64 mg, 0.1 mmol), 1.0 equiv of HON^Me^ (28 mg, 0.1 mmol), and [Cp_2_Fe][PF_6_] (33 mg, 0.1 mmol) were added into the Schlenk tube containing 3 mL of THF under a N_2_ atmosphere at room temperature. After the reaction solution was stirred for 1 h and then stood for 30 min, the top layer THF solution was detected by IR, as shown in Figure S6. IR ν_NO_ 1792 s, 1779 s, 1721 s, 1716 vs cm^–1^ (THF) demonstrated the simultaneous formation of complexes **3** and **4** upon the reaction of complex **1** and [Cp_2_Fe][PF_6_] in the presence of HON^Me^ ligand, suggesting the release of {Fe(NO)_2_}^10^ unit captured by HON^Me^ yielding complex **4**.

### 4.8 Reduction of Complex **3**

THF solution (3 mL) of complex **3** (34 mg, 0.05 mmol) was added into a Schlenk tube containing 1.0 equiv of KC_8_ (7 mg) + 18-crown-6-ether (13 mg) under a N_2_ atmosphere at ambient temperature. The reaction solution was stirred for 1 h, and the mixture solution was observed to convert into green solution. IR ν_NO_ stretching frequencies 1775 s, 1716 vs, 1668 vs cm^–1^ (THF) suggested the formation of the known dinuclear DNIC **1**, as shown in Figure S9. In a similar fashion, 2.0 equiv of KC_8_ + 18-crown-6-ether added into the THF solution (3 mL) of complex **3** (34 mg, 0.05 mmol) led to the formation of red solution monitored by IR. The appearance of IR ν_NO_ stretching frequencies 1663 s, 1611 vs cm^–1^ (THF) implicated the formation of DNIC **2**, as shown in Figure S9.

### 4.9 Preparation of [(μ-ON^Me^)_2_Fe_2_(NO)_4_] (**4**)

A THF solution (15 mL) of [Fe(CO)_2_(NO)_2_] (0.5 mmol) prepared freshly was added to the 25 mL Schlenk flask containing 4-*tert*-butyl-2-(2,6-dimethylphenyliminomethyl)phenol (HON^Me^) (140 mg, 0.5 mmol) by cannula under N_2_. The reaction mixture was allowed to stir for 1 day at ambient temperature. The resulting brown solution was dried under vacuum, and *n*-hexane (30 mL) was then added to dissolve the residue. The *n*-hexane solution was dried under vacuum to obtain the reddish-brown powder [(μ-ON^Me^)_2_Fe_2_(NO)_4_] (**4**) (∼143 mg, 72%). The suitable crystals of complex **4** for X-ray crystallography were obtained from evaporation of hexane solution of complex **4**. IR ν_NO_ 1779 s, 1716 s cm^–1^ (THF). Absorption spectrum (THF) λ_max_/nm (ε/M^–1^ cm^–1^): 340 (8800), 400 sh (3750), 500 br (570). Anal. calcd for C_19_H_22_FeN_3_O_3_: C, 57.59; H, 5.60; N, 10.06. Found: C, 58.61; H, 5.64; N, 9.57.

### 4.10 Preparation of [K-18-Crown-6-ether][(ON^Me^)Fe(NO)_2_] (**5**)

Complex **4** (80 mg, 0.2 mmol), KC_8_ (28 mg, 0.2 mmol), and 18-crown-6-ether (53 mg, 0.2 mmol) were added into a Schlenk tube containing 5 mL of THF under a N_2_ atmosphere at 0 °C, and the mixture solution was stirred for 2 h. The resulting reddish brown solution was precipitated by diethyl ether, and the residue was dried under vacuum to obtain the reddish brown solid [K-18-crown-6-ether][(ON^Me^)Fe(NO)_2_] (**5**) (isolated yield 118 mg (84%)). The THF solution of complex **5** was layered with diethyl ether (15 mL) at room temperature for 1 week to obtain dark-red crystals suitable for X-ray crystallography. IR ν_NO_ 1658 s, 1605 vs cm^–1^ (THF). Absorption spectrum (THF) [λ_max_, nm (ε, M^–1^ cm^–1^)]: 310 (8200), 390 (2600), 480 br (410). Anal. calcd for C_31_H_46_FeKN_3_O_9_: C, 53.22; H, 5.59; N, 6.01. Found: C, 52.87; H, 5.96; N, 5.28.

### 4.11 Qualitative Analysis of the Released NO/N_2_O upon the Reduction and Oxidation of Complex **1**

Quantification of N_2_O was performed using Shimadzu GC-2030 gas chromatography equipped with a barrier discharge ionization detector (BID) and Shin Carbon ST (100/120 mesh) columns filled with poly(dimethyl)siloxane (stationary phase, Rtx-1). Helium was adopted as the mobile phase to conduct sample separation. The oven temperature was kept at 40 °C and then raised up to 240 °C (rate 20 °C/min), and the detector was heated to 280 °C. Identification of the released NO and N_2_O is based on gas chromatogram with retention times of 2.35 and 8.76 min (Figures S2 and S5), respectively.

### 4.12 Crystallography

The single-crystal X-ray crystallographic data collections for complexes **1**, **3**, and **5** were carried out at 200 K with a Bruker D8 Venture Dual X-ray Single-Crystal Diffractometer with graphite-monochromated Mo-Kα radiation (λ = 0.71073 Å) outfitted with a low-temperature, nitrogen-stream aperture. Single crystals of **2** and **4** kept at 99.99(10) K during data collection were collected on a XtaLAB Synergy R, DW system, HyPix-Arc 150 diffractometer with graphite-monochromated Cu–Kα radiation (λ = 1.54184 Å) outfitted with a low-temperature, nitrogen-stream aperture. Both structures were solved by direct methods, in conjunction with standard difference Fourier techniques, and refined by full-matrix least-squares procedures. Least-squares refinement of the positional and anisotropic thermal parameters of all non-hydrogen atoms and fixed hydrogen atoms were refined by the riding model.^86^ The SHELXTL structure refinement program was employed.^87^ A summary of the crystallographic parameters for complexes **1**–**5** is shown in Tables S1–S3.

### 4.13 EPR Measurements

The X-band EPR measurements were recorded on a Bruker E580 CW/Pulse EPR equipped with an Oxford liquid He quartz cryostat. The EPR samples (THF solution) of complexes **1**–**5** were transferred to J. Young valved EPR tubes. At 298 K, X-band EPR spectra were obtained with a frequency of 9.484 GHz. The microwave power and modulation amplitude are 15.0 mW and 0.8 G at 100.00 kHz, respectively. At 77 and 10 K, X-band EPR spectra were obtained with the microwave power of 15 mW, a frequency of 9.484 GHz, and a modulation amplitude of 16 G at 100.00 kHz. EPR spectra were simulated using EasySpin software.^88^ Additionally, the spin counting of complex **1** was conducted at room temperature using spin-only {Fe(NO)_2_}^9^ [PPN][Fe(NO)_2_(SPh)_2_] (*S* = 1/2) as spin reference. The calibration curve for spin counting was established from a stock solution with various concentrations of [PPN][Fe(NO)_2_(SPh)_2_] dissolved in dry THF under a N_2_ atmosphere; the details are given in the Supporting Information.^79−83,89,90^

### 4.14 Magnetic Measurements

The magnetic data of the microcrystalline samples of complexes **1**–**5** were placed in a polycarbonate capsule and were recorded using a SQUID magnetometer (SQUID-VSM, Quantum Design) under an external magnetic field (0.5 T) in the temperature range of 2–300 K. The magnetic susceptibility data were corrected with ligands’ diamagnetism by the tabulated Pascal’s constants.^91,92^ These analysis were performed by taking molar magnetic susceptibility (χ_m_) as a function of the absolute temperature (*T*) and fitting the data to Boltzmann distribution.

### 4.15 XAS Measurements

All Fe K-edge spectra were completed at the National Synchrotron Radiation Research Center (NSRRC), Hsinchu, Taiwan. The measurements were carried out at the TPS 44A beamline (quick-scanning X-ray absorption spectroscopy, 4.5–34 keV) with a double crystal Si(111) monochromator and were recorded at room temperature. The energy resolution Δ*E*/*E* was estimated to be about 2 × 10^–4^. High harmonics were rejected by Rh-coated mirrors. Ion chambers used to measure the incident (*I*_0_) and transmitted (*I*) beam intensities were filled with a mixture of N_2_ and He gases and a mixture of N_2_ and Ar gases, respectively. The spectra were scanned from 6.912 to 8.006 keV. The energy calibration was referred to by measuring, simultaneously, iron foil (the first inflection point at 7112.0 eV). The energy resolution of 0.3 eV can be achieved. A smooth background was removed from all spectra by fitting a straight line to the pre-edge region and then subtracting this straight line from the entire spectrum. Normalization of the data was accomplished by fitting a flat polynomial to the postregion and also normalizing the edge jump to 1.0 at 7400 eV for Fe K-edge spectra.^93−95^

## Supporting Information Available

Details of spectroscopic data (FTIR, EPR, CV, SQUID, and XAS) and X-ray diffraction data collection details (DOCX)
