# Supplementary material for: Electronic Structure and Transformation of Dinitrosyl Iron Complexes (DNICs) Regulated by Redox Non-Innocent Imino-Substituted Phenoxide Ligand
Source: Inorg Chem. 2024 Jan 23;63(5):2431–42. doi: 10.1021/acs.inorgchem.3c03367 (PMC10848267; doi:10.1021/acs.inorgchem.3c03367)
Supplement: Supplementary file 1 — ic3c03367_si_001.docx [file ic3c03367_si_001.docx]

**Supporting Information**

**Electronic Structure and Transformation of Dinitrosyl Iron Complexes (DNICs) Regulated by Redox Non-Innocent Imino-substituted Phenoxide Ligand**

Wun-Yan Wu,*^a^* Wei-Yuan Zheng, *^a^***^∥^** Wei-Ting Chen, *^a^***^∥^** Fu-Te Tsai,^*^*^a^* Ming-Li Tsai,*^b^* Chih-Wen Pao, *^c^* Jeng-Lung Chen, *^c^* Wen-Feng Liaw^*^*^a^*

*^a^*Department of Chemistry, National Tsing Hua University, Hsinchu 30013, Taiwan

*^b^*Department of Chemistry, National Sun Yat-sen University, Kaohsiung 80424, Taiwan

*^c^*National Synchrotron Radiation Research Center, Hsinchu 30013, Taiwan

E-mail address of the corresponding authors:

[fttsai@mx.nthu.edu.tw](mailto:fttsai@mx.nthu.edu.tw) (FTT); [wfliaw@mx.nthu.edu.tw](mailto:wfliaw@mx.nthu.edu.tw) (WFL)

**

**

**Figure S1.** X-band EPR spectra (solid) of [Fe(NO)_2_(*μ*-ON_2_^Me^)Fe(NO)_2_] (**1**) with g = 2.008 at room temperature. (9.484191 GHz, Power = 15 mW, receiver gain = 30).





**Figure S2.** Fe K-edge XAS of complexes **1-5**. Inset plot: the pre-edge energy of complexes **1**-**5** (**1** at 7113.6 eV, **2** at 7113.6 eV, **3** at 7114.0 eV, **4** at 7113.8 eV, **5** at 7113.7 eV; the standard of Fe^II^ and Fe^III^ complexes were based on the previously reported data, respectively).

**Spin counting of DNIC 1**

All spin counting measurements and calibration samples were conducted at room temperature utilizing the mononuclear {Fe(NO)_2_}^9^ DNIC. The spin counting calibration curve was established using [PPN][Fe(NO)_2_(SPh)_2_] (S = 1/2) in calibration samples derived from a stock solution with various concentrations of [PPN][Fe(NO)_2_(SPh)_2_] in dry THF under N_2_ atmosphere. The stock solution of [PPN][Fe(NO)_2_(SPh)_2_] (17.5 mg, 2 mL, 10.00 mM) was appropriately diluted to generate samples with concentrations of 1.00 mM and 0.50 mM. For all measurements, a single 4 mm outer diameter X-band (9.484191 GHz) quartz tube was employed and filled with 200 mL solution for each calibration measurement. The tube position was marked to maintain consistent height and orientation throughout the calibration measurements, ensuring the sample's center aligned with the resonator cavity window's center. Conditions: Field Mod. Amplitude = 0.00016 T, Field Mod. Frequency = 100000 Hz, Microwave Frequency = 9.484191 GHz, Microwave Power = 1.5 mW, Receiver Gain = 20. Experimental spectra underwent baseline correction before integration. The resulting absorption spectra underwent a secondary baseline correction was applied to determine the second integral spectral intensity. Spin quantification of complex **1** (1 mM in THF, respectively) were followed by the same data treatment to obtain the relative spin concentration (93.78%) versus calibrated [Fe(NO)_2_(SPh)_2_]^-^ spectra.





**Figure S3.** (a) EPR spectra of internal standard [PPN][Fe(NO)_2_(SPh)_2_]. (b) EPR spectrum of 1.0 mM complex **1** in THF at 298 K. (298 K, 9.484191 GHz, Power = 1.5 mW, receiver gain = 20). (c) Spin quantification relies on known concentrations (1.00 mM, 0.50 mM) of the internal standard [PPN][Fe(NO)_2_(SPh)_2_] (S = 1/2), utilizing its second integral spectral intensity for interpolation.


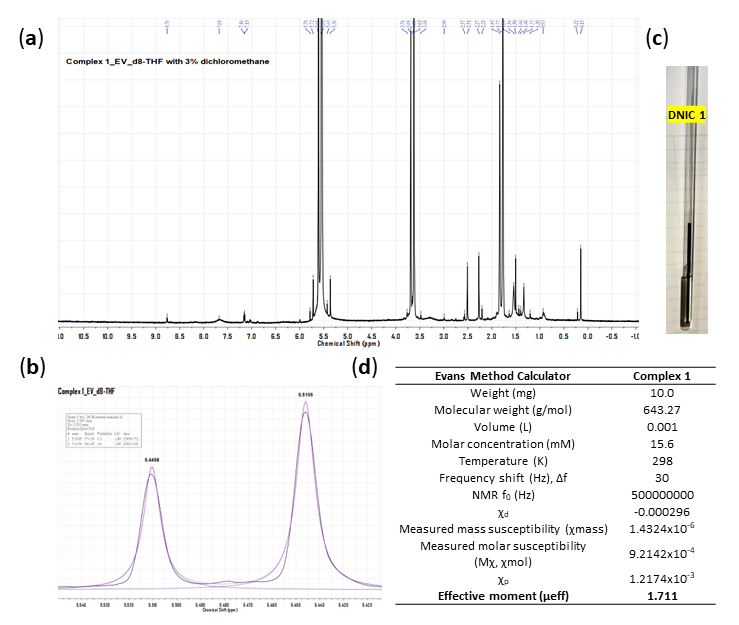


**Figure S4.** (a) Magnetic measurements of the *d*^8^-THF solution of complex **1** with 3% dichloromethane (δ 5.545 ppm) performed in 500 MHz ^1^H-NMR at 298 K. (b) Evans method: the peak fitting of internal standard shift present in **1**. (c) experimental scheme and (d) magnetic calculation data table for complex **1** is shown above.





**Figure S5.** Detection of NO and N_2_O derived from DNIC **1** + KC_8_ + 18-crown-6-ether, based on gas chromatogram with retention time at 2.7 and 8.2 min, respectively. (Note: The generation of NO_(g)_ may be consumed by O_2(g)_ from sampling.)





**Figure S6.** X-band EPR spectra (solid) of [K-18-crown-6-ether)][(ON_2_^Me^)Fe(NO)_2_] (**2**) with g_1_ = 2.034, g_1_ = 2.013 and g_1_ = 2.004 at at room temperature. (9.484191 GHz, Power = 15 mW, receiver gain = 30)

**
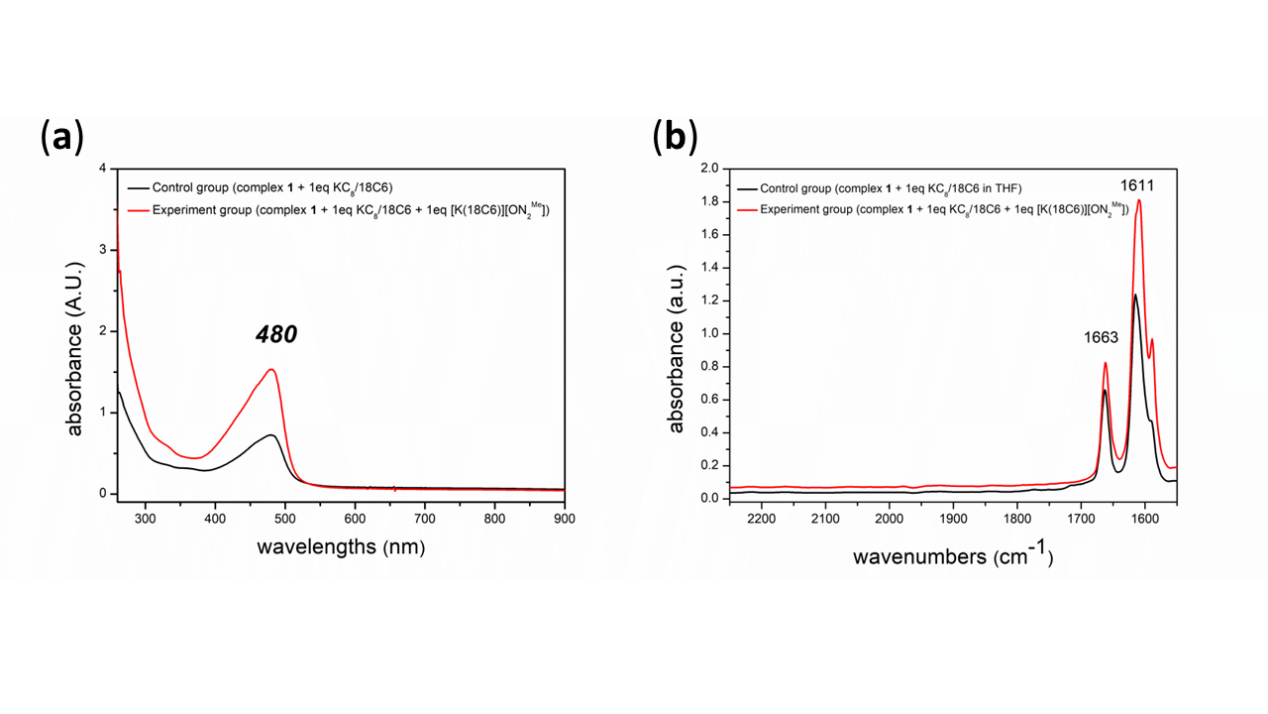
**

**Figure S7.** (a) UV-vis and (b) IR spectra for the reaction of DNIC **1** and [KC_8_]+[18-crown-6-ether] with (red line) and without (black line) the presence of [K-18-crown-6-ether][ON_2_^Me^] in THF.


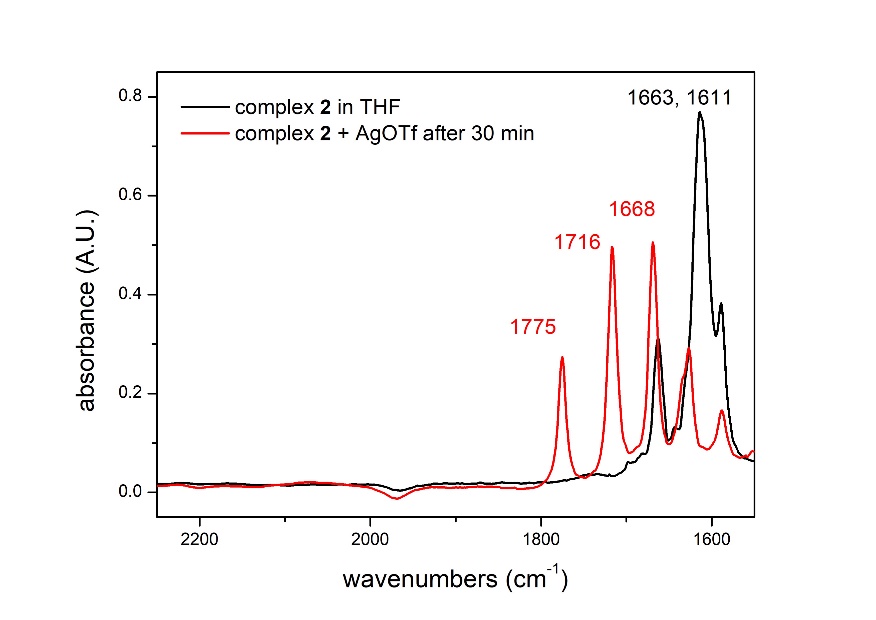


**Figure S8.** IR spectra (THF) for the reaction of 0.5 equiv of sliver triflate (AgOTf) and [K-18-crown-6-ether)][(ON_2_^Me^)Fe(NO)_2_] (**2**).





**Figure S9.** Detection of NO and N_2_O derived from DNIC **1** + [FeCp_2_][PF_6_], based on gas chromatogram with retention time at 2.7 and 8.2 min, respectively.


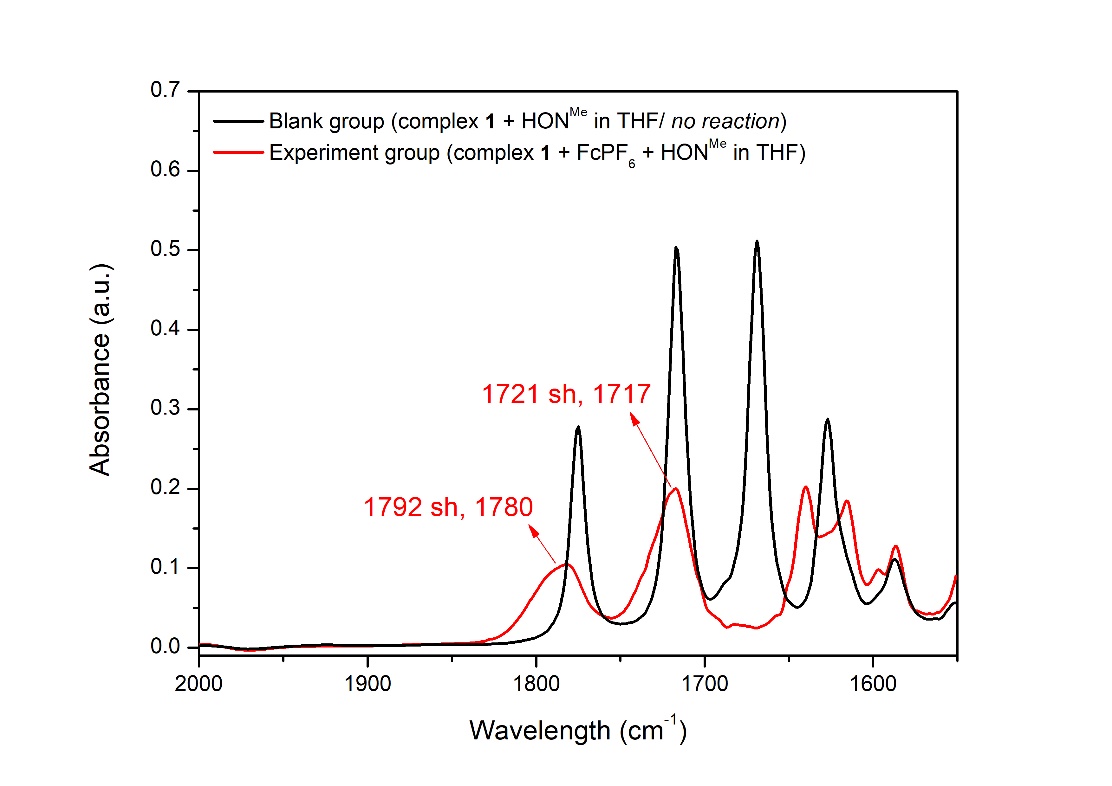


**Figure S10.** IR spectra (THF) for the reaction of DNIC **1** + 1.0 equiv of HON^Me^ (black line) and the reaction of complex **1** + 1.0 equiv of [Cp_2_Fe][PF_6_] + 1.0 equiv of HON^Me^ (red line).


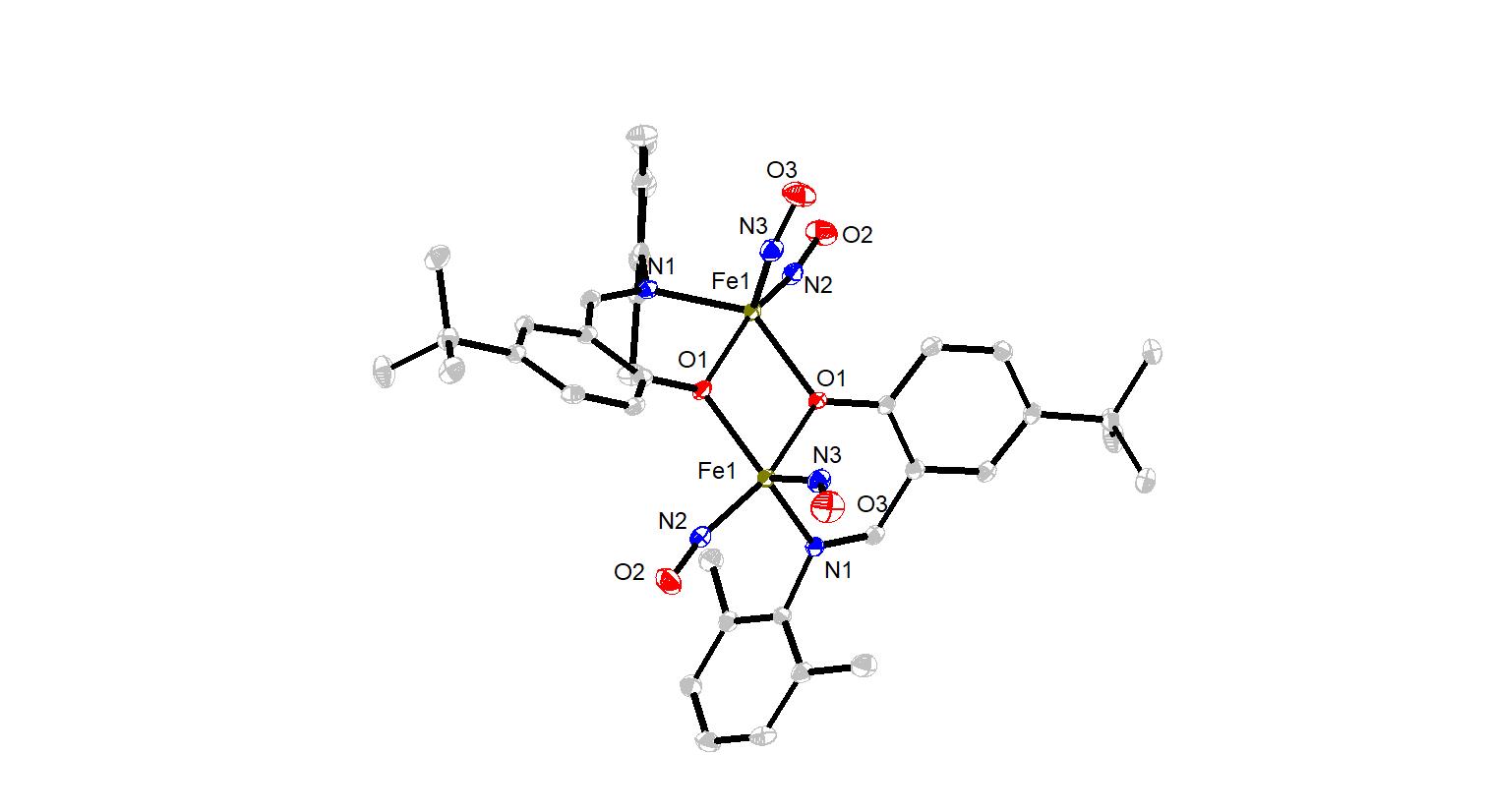


**Figure 11.** Crystallographic structure of [(***μ*-**ON^Me^)_2_Fe_2_(NO)_4_] (**4**). Selected bond lengths and bond angles are shown in Table S1.


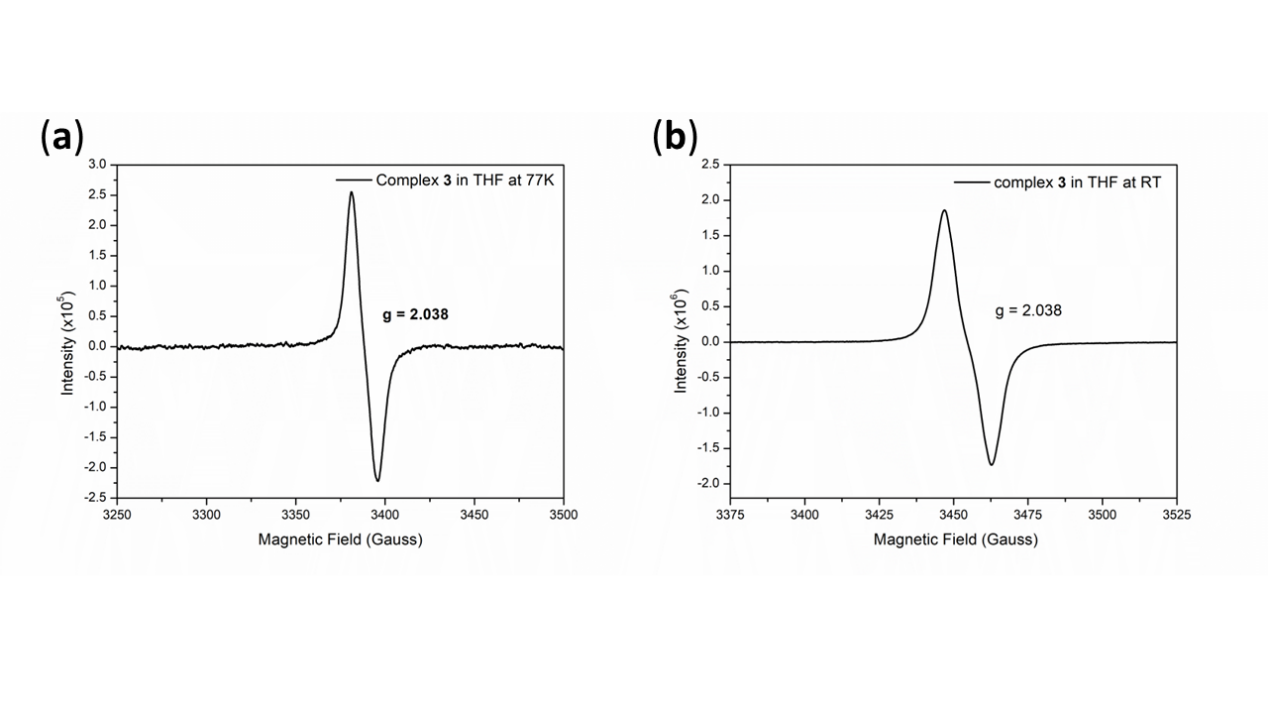


**Figure S12.** X-band EPR spectra (THF) of [(ON_2_^Me^)Fe(NO)_2_][PF_6_] (**3**) (a) with g = 2.038 at 77 K and (b) with g_av_ = 2.038 at room temperature.


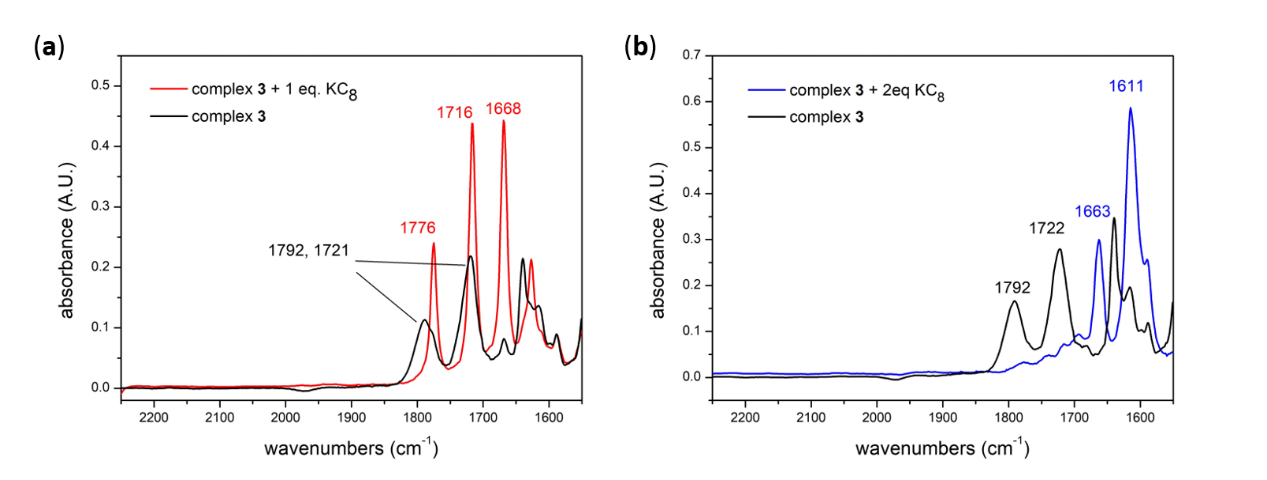


**Figure S13.** IR spectra (THF) for the reaction of [(ON_2_^Me^)Fe(NO)_2_][PF_6_] (**3**) with (a) 1.0 equiv of KC_8_ (red line) and (b) 2.0 equiv of KC_8_ (blue line), respectively.





**Figure S14.** Cyclic voltammogram of 3 mM complex **3** measured in a THF solution with 0.2 M [n-Bu_4_N][PF_6_] as the supporting electrolyte (ferrocene as the internal standard at room temperature, scan rate 0.025 V/s to 1.0 V/s).

**

**

**Figure S15.** Cyclic voltammogram of 3 mM complex **3** measured in a THF solution with 0.2 M [n-Bu_4_N][PF_6_] as the supporting electrolyte (ferrocene as the internal standard at room temperature, scan rate 0.025 V/s to 1.0 V/s).


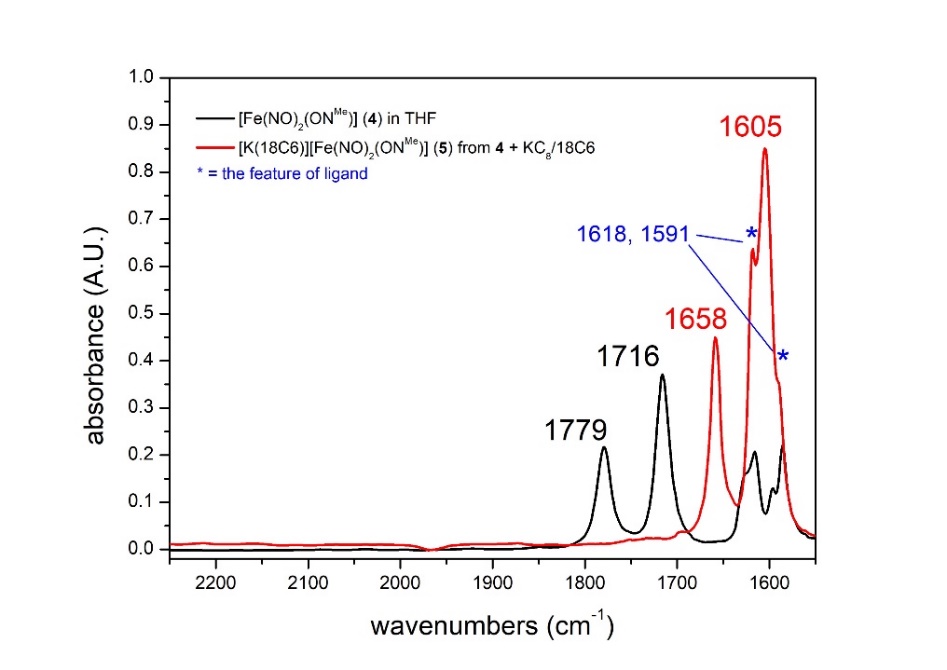


**Figure S16.** IR spectra (THF) of [(*μ***-**ON^Me^)_2_Fe_2_(NO)_4_] (**4**) (black line) and [K-18-crown-6-ether][(ON^Me^)Fe(NO)_2_] (**5**) (red line).

**
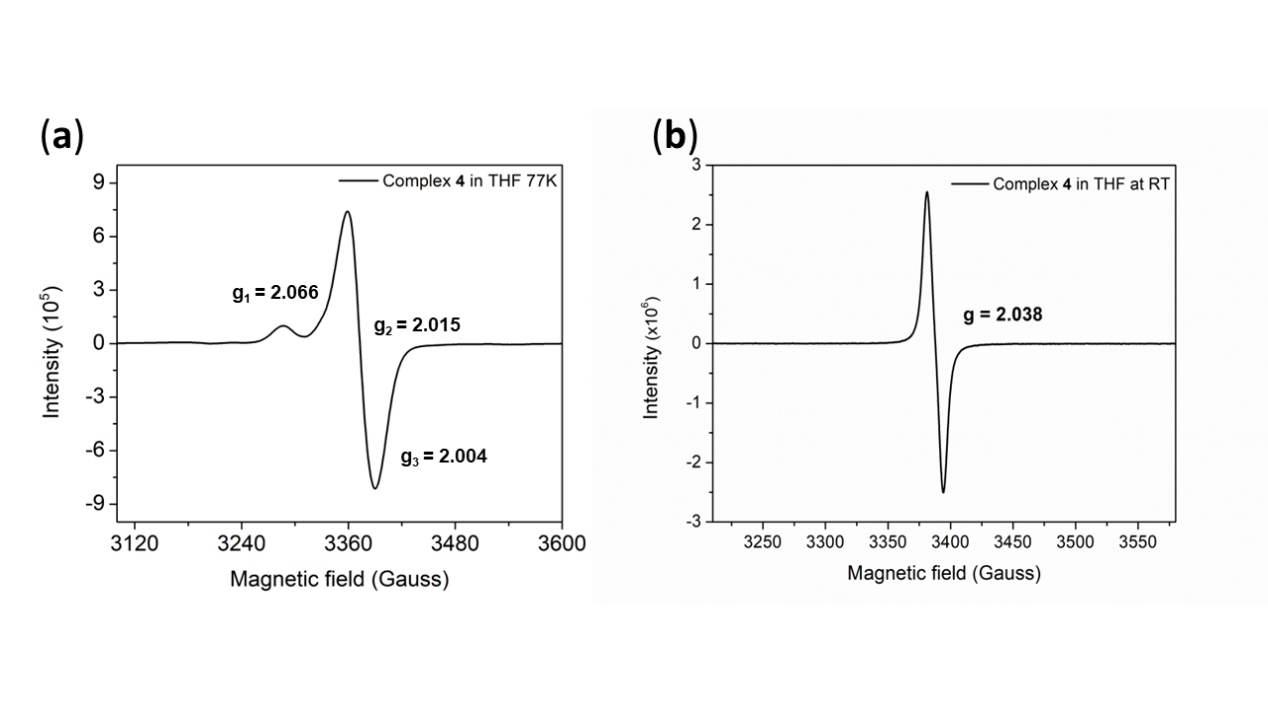
**

**Figure S17.** X-band EPR spectra of [(*μ***-**ON^Me^)_2_Fe_2_(NO)_4_] (**4**) (a) with g_1_ = 2.066, g_2_ = 2.015, g_3_ = 2.004 at 77 K; (b) with g = 2.038 at room temperature.


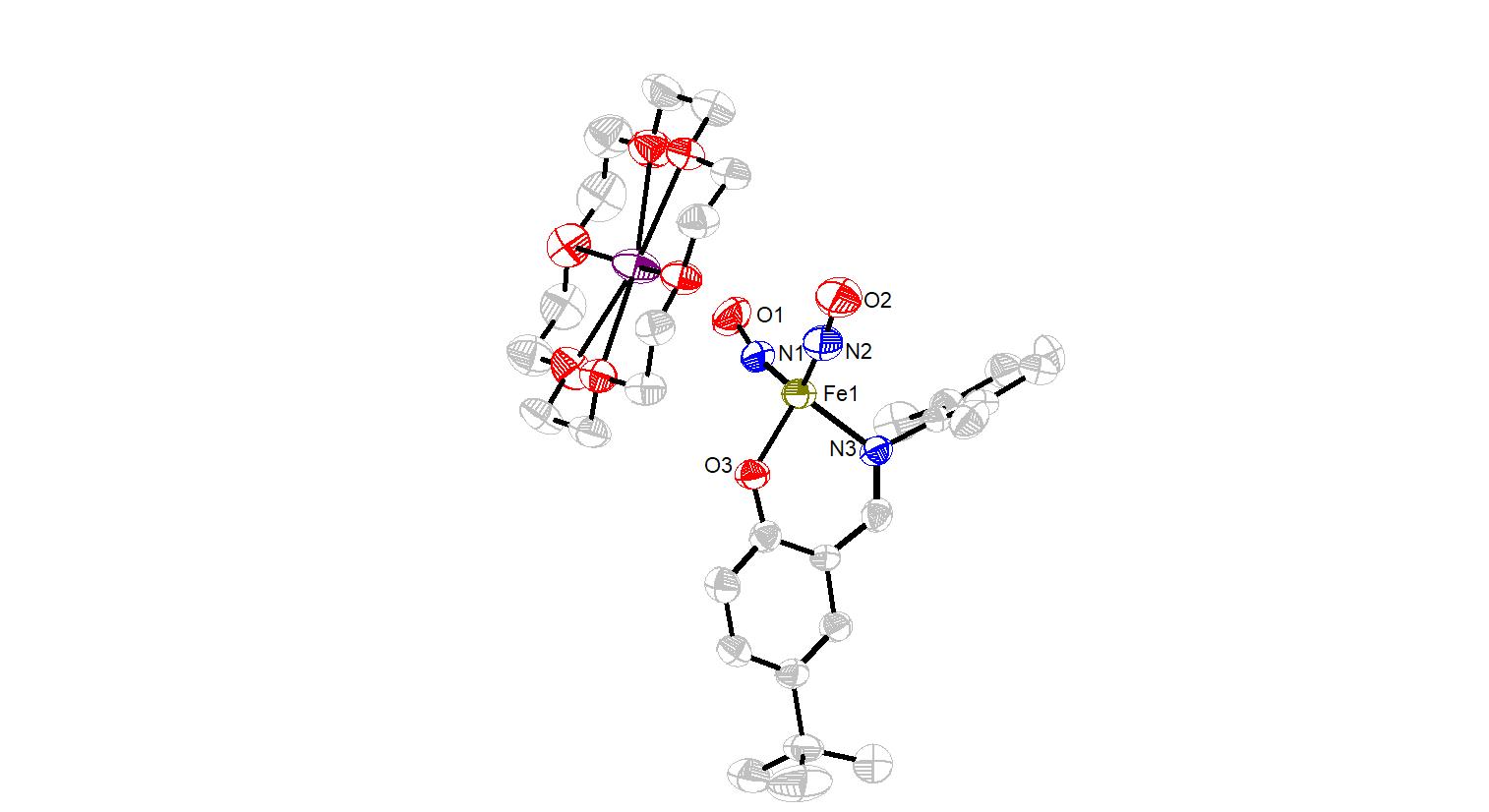


**Figure S18.** Crystallographic structure of [K-18-crown-6-ether][(ON^Me^)Fe(NO)_2_] (**5**). Selected bond lengths and bond angles are shown in Table S1.





**Figure S19.** X-band EPR spectra (solid) of [K-18-crown-6-ether)][(ON^Me^)Fe(NO)_2_] (**5**) with g_1_ = 2.034, g_1_ = 2.013 and g_1_ = 2.004 at at room temperature. (9.484191 GHz, Power = 15 mW, receiver gain = 30)

1.
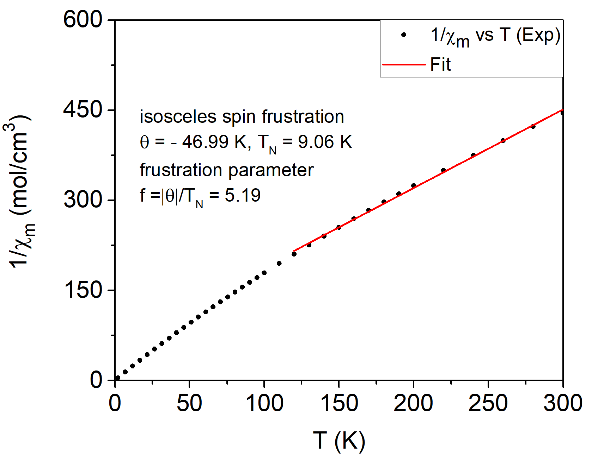
 (b)


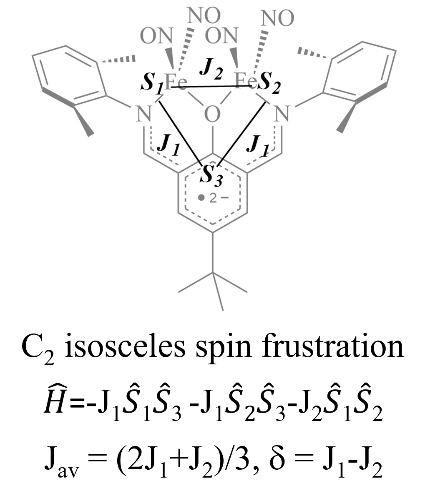


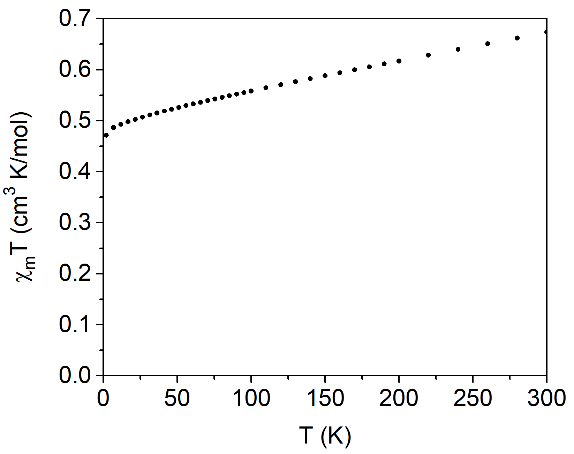

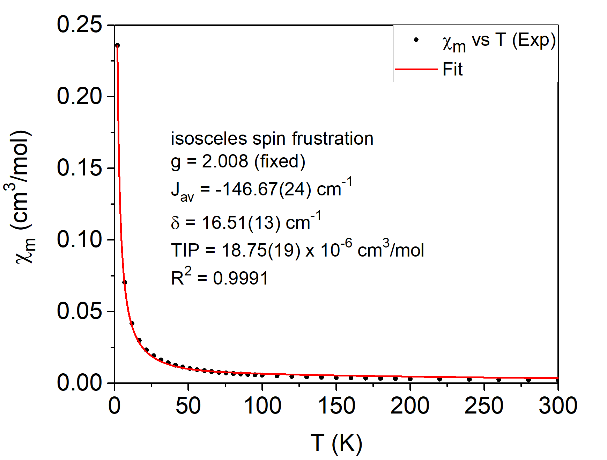
(c)


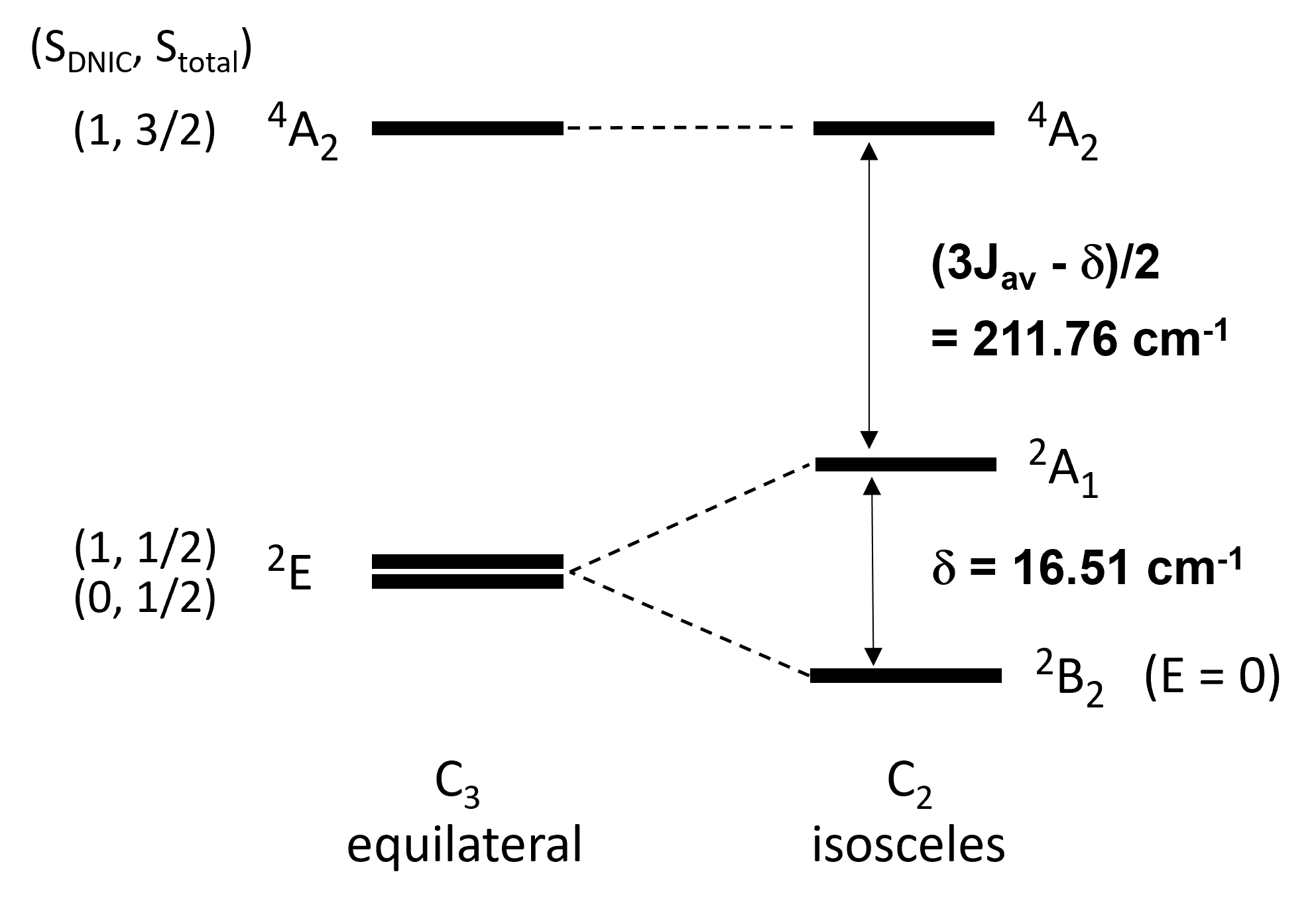
(d)

**Figure S20.** (a) The isotropic magnetic coupling among [^•^ON_2_^Me^]^2−^ and the adjacent two {Fe(NO)_2_}^9^ cores in DNIC **1**. (b) Curie-Weiss fitting of 1/χ_m_ vs T plot above 120 K to determine frustration parameter. (c) The best fitting of χ_m_ vs T and χ_m_T vs T plots. (d) Energy diagram of magnetic states for [{Fe(NO)_2_}^9^-{Fe(NO)_2_}^9^-[^•^ON_2_^Me^]^2−^] electronic structure in DNIC **1**, showing that the C_2_-induced mixing of (0, 1/2) and (1, 1/2) magnetic states results in ^2^A_1_ and ^2^B_2_ doublet states.


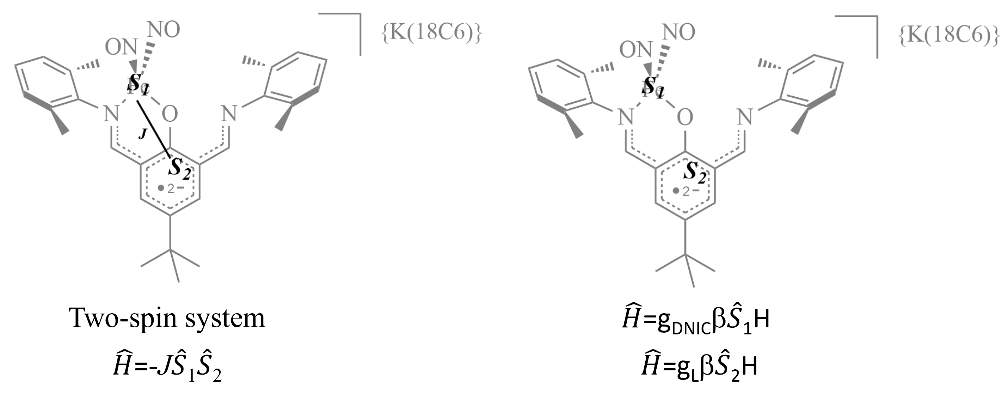
(a)

(b)


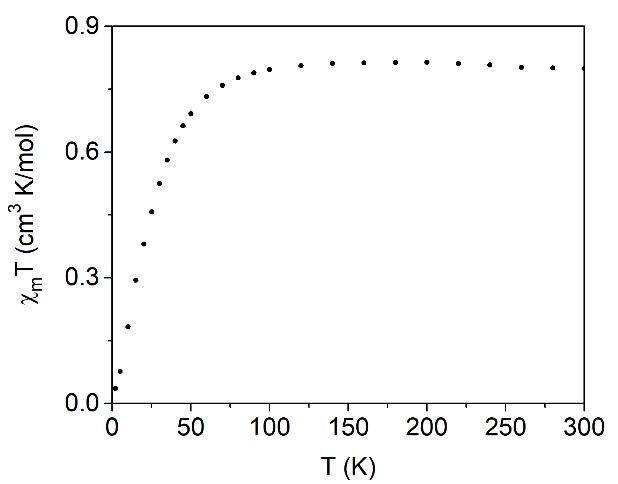

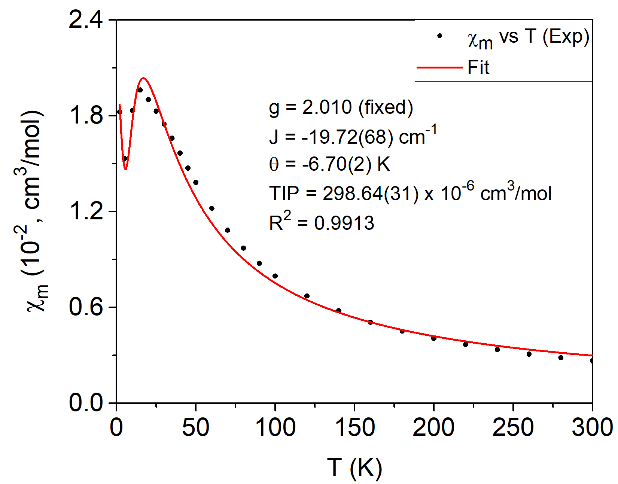


**Figure S21.** (a) The magnetic coupling between [^•^ON_2_^Me^]^2−^ and the adjacent {Fe(NO)_2_}^9^ core in DNIC **2**. (b) The best fitting of molar magnetic susceptibility (χ_m_) vs absolute temperature (T) and χ_m_T vs T plot.


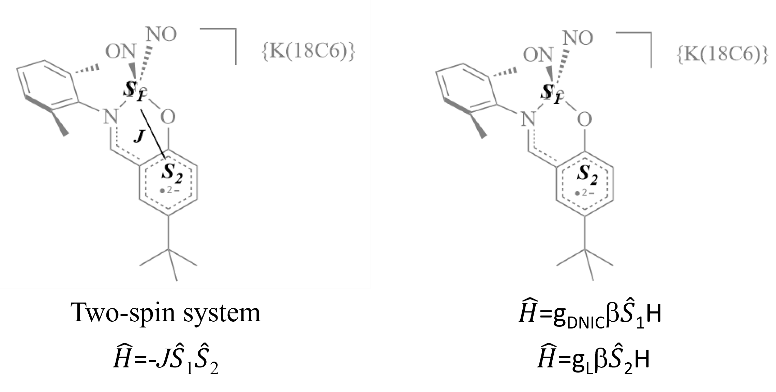
(a)

(b)


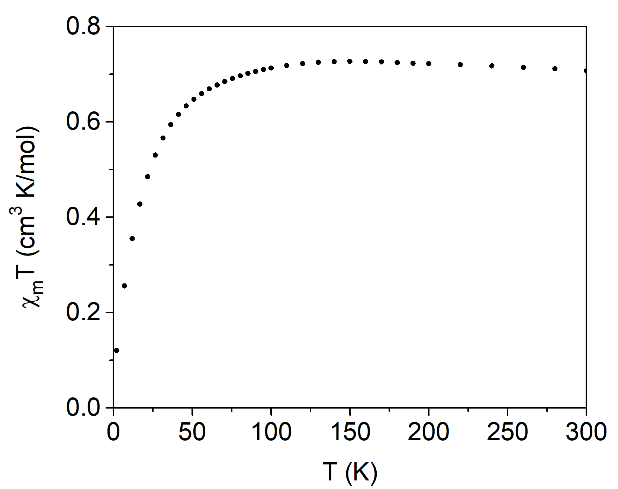

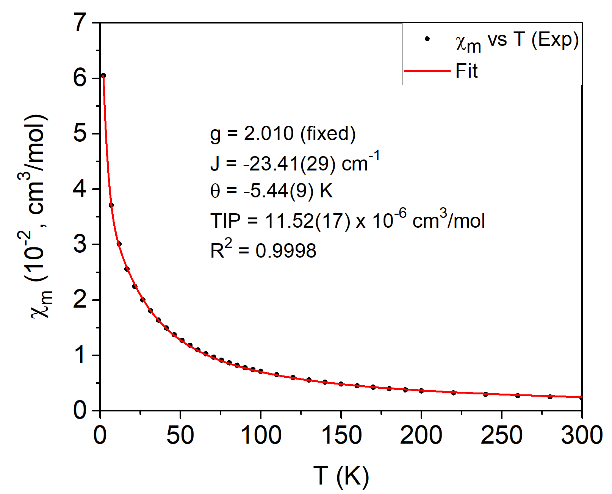


**Figure S22.** (a) The magnetic coupling between [^•^ON^Me^]^2−^ and the adjacent {Fe(NO)_2_}^9^ core in DNIC **5**. (b) The best fitting of molar magnetic susceptibility (χ_m_) vs absolute temperature (T) and χ_m_T vs T plot.


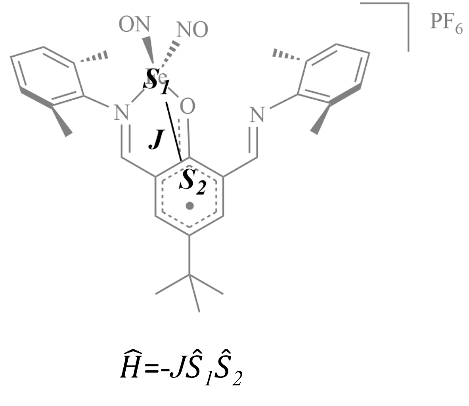
(**a**)


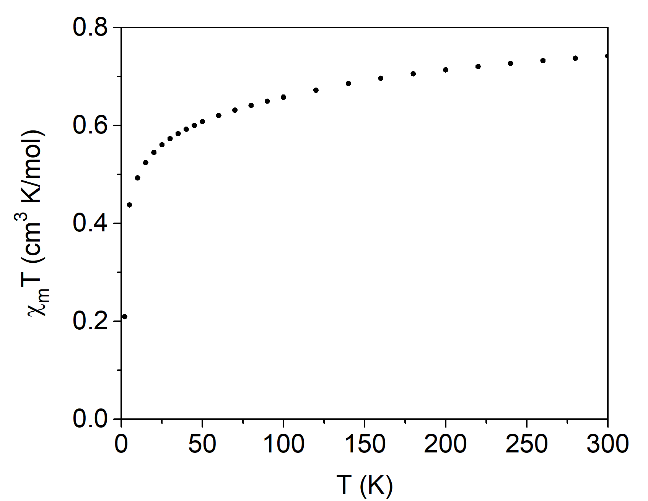

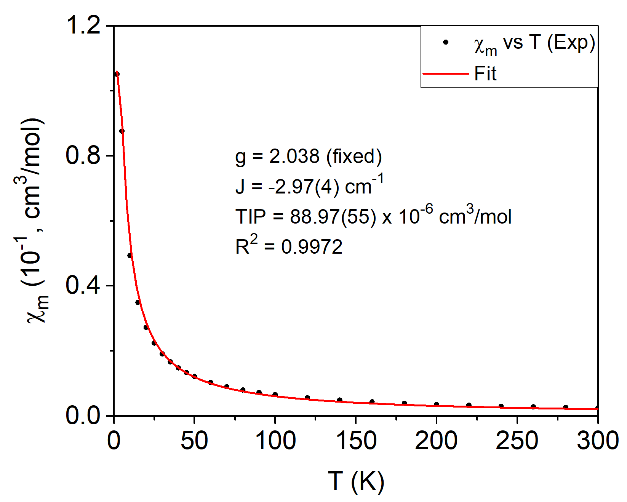
(**b**)

**Figure S23.** (a) The magnetic coupling between [^•^ON_2_^Me^] phenoxyl radical and the adjacent {Fe(NO)_2_}^9^ core in DNIC **3**. (b) The best fitting of molar magnetic susceptibility (χ_m_) vs absolute temperature (T) and χ_m_T vs T plot.


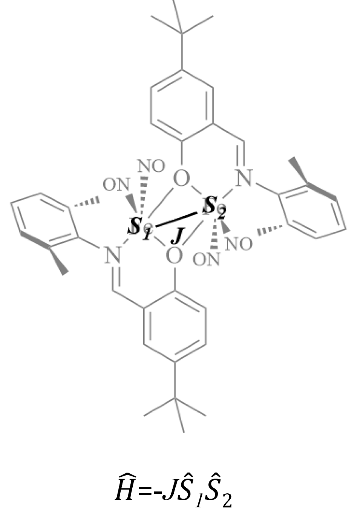
(**a**)


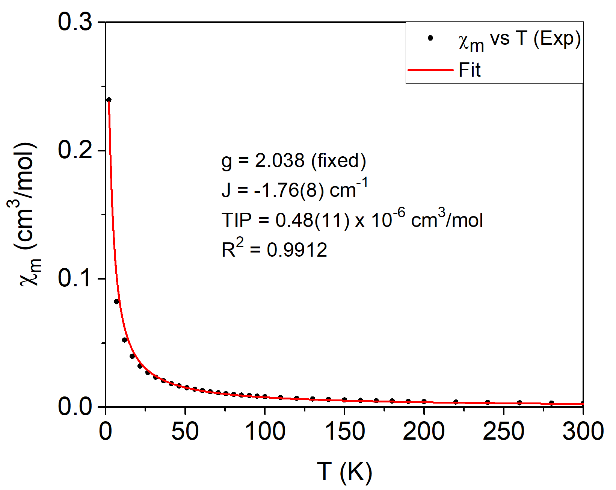

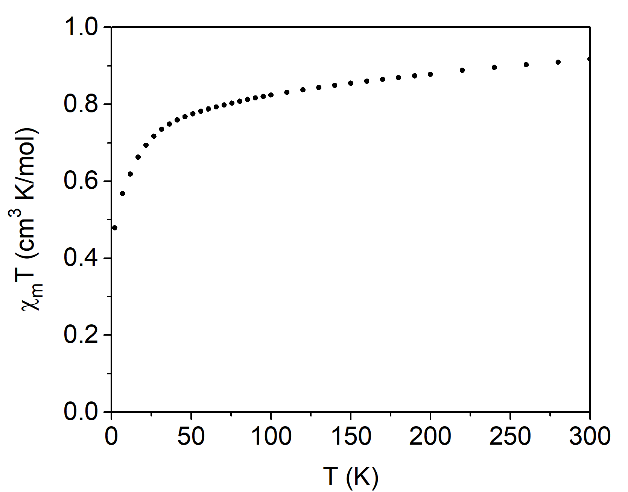
(**b**)

**Figure S24.** (a) The magnetic coupling of the adjacent two {Fe(NO)_2_}^9^ cores in DNIC **4**. (b) The best fitting of molar magnetic susceptibility (χ_m_) vs absolute temperature (T) and χ_m_T vs T plot.

**Table S1.** Selected bond lengths (Å) and angles (deg) for crystallographic structures of complexes **1**-**5**.

|  | **1** | **2** | **3** | **4** | **5** |
| --- | --- | --- | --- | --- | --- |
| **Bond lengths (Å)** | | | | | |
| Fe(1)···Fe(2) | 2.7394(6) | N/A | N/A | 3.366 (Fe1^…^Fe1’) | N/A |
| Fe(1)−N(1) | 1.663(3) | 1.644(2) | 1.674(6) | 1.711(2) | 1.649(5) |
| Fe(1)−N(2) | 1.665(3) | 1.645(2) | 1.683(6) | 1.730(2) | 1.650(5) |
| Fe(2)−N(3) | 1.668(3) | N/A | N/A | N/A | N/A |
| Fe(2)−N(4) | 1.674(3) | N/A | N/A | N/A | N/A |
| **Average Fe−N(O)** | **1.6675(3)** | **1.6445(2)** | **1.6785(6)** | **1.7205(2)** | **1.6495(5)** |
| N(1)−O(1) | 1.190(3) | 1.217(2) (N(1)-O(2)) | 1.177(7) | N/A | 1.199(5) |
| N(2)−O(2) | 1.190(3) | 1.223(3) (N(1)-O(2)) | 1.151(7) | 1.168(2) | 1.194(5) |
| N(3)−O(3) | 1.179(3) | N/A | N/A | 1.189(2) | N/A |
| N(4)−O(4) | 1.179(3) | N/A | N/A | N/A | N/A |
| **Average N−O** | **1.1845(3)** | **1.220(2)** | **1.164(7)** | **1.178(2)** | **1.1965(5)** |
| Fe−O_phenol_ | 1.979(2)/1.975(2) | 1.9985(15) | 1.931(4) | 2.0768(11) | 1.992(4) |
| O−C_phenol_ | 1.313(3) | 1.299(3) | 1.300(6) | 1.333(2) | 1.299(6) |
| **Bond angles (deg)** | | | | | |
| Fe(1)−O_R_−Fe(2) | 87.71(7) | N/A | N/A | 108.36(5) | N/A |
|  |  |  |  |  |  |
| O(1)−N(1)−Fe(1) | 168.7(2) | 157.5(2) (O(2)-N(1)-Fe(1)) | 157.9(6) | N/A | 164.5(5) |
| O(2)−N(2)−Fe(1) | 169.4(3) | 158.7(2)(O(3)-N(2)-Fe(2)) | 161.0(7) | N/A | 167.8(5) |
| O(3)−N(3)−Fe(2) | 162.1(3) | N/A | N/A | 161.50(13) | N/A |
| O(4)−N(4)−Fe(2) | 163.5(3) | N/A | N/A | 145.75(13) | N/A |
| **Average O−N−Fe** | **165.9(3)** | **157.1(5)** | **159.5(6)** | **153.62(13)** | **166.15(5)** |

| Complex number | 1 | 2 | 3 |
| --- | --- | --- | --- |
| Empirical formula | C_28_H_31_Fe_2_N_6_O_5_ | C_40_H_55_FeKN_4_O_9_ | C_32_H_39_F_6_FeN_4_O_4_P |
| Crystal size (mm3) | 0.24 x 0.09 x 0.01 | 0.15 x 0.15 x 0.11 | 0.19 x 0.03 x 0.02 |
| Formula mass | 643.29 | 830.83 | 744.49 |
| Crystal system | Monoclinic | Monoclinic | Orthorhombic |
| *a*/Å | 9.8116(17) | 16.5334(4) | 11.9159(6) |
| *b*/Å | 6.9718(11) | 18.9874(4) | 16.9876(11) |
| *c*/Å | 43.539(7) | 15.6729(4) | 35.326(2) |
| α/° | 90 | 90 | 90 |
| β/° | 96.354(3) | 108.384(2) | 90 |
| γ/° | 90 | 90 | 90 |
| Unit cell volume/Å^3^ | 2960.0(8) | 4669.0(2) | 7150.7(7) |
| Temperature/K | 200(2) | 99.99(10) | 200(2) |
| Space group | P 2_1/n_ | P 2_1/c_ | Pbca |
| No. of formula units per unit cell, Z | 4 | 4 | 8 |
| Radiation type | Mo K_α_ | Cu Kα | Mo K_α_ |
| Density (calculated), Mg/m^3^ | 1.444 | 1.182 | 1.383 |
| Absorption coefficient, mm^-1^ | 1.027 | 3.809 | 0.538 |
| F(000) | 1332 | 1760 | 3088 |
| Reflections collected | 23232 | 34631 | 35574 |
| Independent reflections | 5253 [R(int) = 0.0520] | 8796 [R(int) = 0.0448] | 6314 [R(int) = 0.0920] |
| Data / restraints / parameters | 5253 / 0 / 377 | 8796 / 0 / 503 | 6314 / 0 / 437 |
| Goodness-of-fit on F^2^ | 1.009 | 1.078 | 1.004 |
| Final R indices [I>2sigma(I)] | R1 = 0.0389  wR2 = 0.1053 | R1 = 0.0432  wR2 = 0.1153 | R1 = 0.0782  wR2 = 0.1961 |
| R indices (all data)^a,b^ | R1 = 0.0522  wR2 = 0.1153 | R1 = 0.0546  wR2 = 0.1203 | R1 = 0.1395  wR2 = 0.2405 |
| Largest diff. peak and hole,e.Å^-3^ | 0.471, -0.280 | 0.30, -0.34 | 0.934, -0.584 |

**Table S2.** Summary of crystallographic data, intensity collection and structure refinement parameters for **1-3**.

^a^ R1 = (Σ||*F*_o_|-|*F*_c_||)/(Σ|*F*_o_|)^b^*w*R2 = [Σ*w*(*F*_o_^2^-*F*_c_^2^)^2^/Σ*w*(*F*_o_^2^)^2^]^1/2^

**Table S3.** Summary of crystallographic data, intensity collection and structure refinement parameters for **4** and **5**.

| Complex number | 4 | 5 |
| --- | --- | --- |
| Empirical formula | C_38_H_44_Fe_2_N_6_O_6_ | C_31_H_46_FeKN_3_O_9_ |
| Crystal size (mm3) | 0.07 x 0.03 x 0.02 | 0.79 x 0.02 x 0.01 |
| Formula mass | 396.24 | 699.66 |
| Crystal system | Monoclinic | Monoclinic |
| *a*/Å | 22.0364(6) | 14.692(6) |
| *b*/Å | 9.8347(2) | 13.252(5) |
| *c*/Å | 19.4963(5) | 21.541(8) |
| α/° | 90 | 90 |
| β/° | 114.065(3) | 108.634(8) |
| γ/° | 90 | 90 |
| Unit cell volume/Å^3^ | 3858(18) | 3974(3) |
| Temperature/K | 99.99(10) | 200(2) |
| Space group | C 2_/c_ | P 2_1/n_ |
| No. of formula units per unit cell, Z | 8 | 4 |
| Radiation type | Cu K_α_ | Mo K_α_ |
| Density (calculated), Mg/m^3^ | 1.364 | 1.169 |
| Absorption coefficient, mm^-1^ | 6.460 | 0.531 |
| F(000) | 1656 | 1480 |
| Reflections collected | 24686 | 54144 |
| Independent reflections | 3699 [R(int) = 0.0344] | 7152 [R(int) = 0.2219] |
| Data / restraints / parameters | 5699 / 0 / 240 | 7152 / 2 / 410 |
| Goodness-of-fit on F^2^ | 1.063 | 0.856 |
| Final R indices [I>2sigma(I)] | R1 = 0.0280  wR2 = 0.0681 | R1 = 0.0693  wR2 = 0.1274 |
| R indices (all data)^a,b^ | R1 = 0.0313  wR2 = 0.0696 | R1 = 0.2149  wR2 = 0.1751 |
| Largest diff. peak and hole,e.Å^-3^ | 0.30, -0.37 | 0.220, -0.226 |

^a^ R1 = (Σ||*F*_o_|-|*F*_c_||)/(Σ|*F*_o_|) ^b^*w*R2 = [Σ*w*(*F*_o_^2^-*F*_c_^2^)^2^/Σ*w*(*F*_o_^2^)^2^]^1/2^
